# Ethnobotanical study on medicinal plants used by Maonan people in China

**DOI:** 10.1186/s13002-015-0019-1

**Published:** 2015-04-30

**Authors:** Liya Hong, Zhiyong Guo, Kunhui Huang, Shanjun Wei, Bo Liu, Shaowu Meng, Chunlin Long

**Affiliations:** College of Life and Environmental Sciences, Minzu University of China, Beijing, 100081 People’s Republic of China; Lineberger Comprehensive Cancer Center, University of North Carolina at Chapel Hill, Chapel Hill, NC 27599 USA; Kunming Institute of Botany, Chinese Academy of Sciences, Kunming, 650201 People’s Republic of China

**Keywords:** Medicinal plants, Traditional knowledge, The Maonans, Ethnomedicine, Huanjiang county

## Abstract

**Background:**

This paper is based on an ethnobotanical investigation that focused on the traditional medicinal plants used by local Maonan people to treat human diseases in Maonan concentration regions. The Maonan people have relied on traditional medicine since ancient times, especially medicinal plants. The aim of this study is to document medicinal plants used by the Maonans and to report the status of medicinal plants and associated traditional knowledge.

**Methods:**

Ethnobotanical data were collected from June 2012 to September 2014 in Huanjiang Maonan Autonomous County, northern Guangxi, southwest China. In total, 118 knowledgeable informants were interviewed. Following statistically sampling method, eighteen villages from 5 townships were selected to conduct field investigations. Information was collected through the approache of participatory observation, semi-structured interviews, ranking exercises, key informant interviews, focus group discussions, and participatory rural appraisals.

**Results:**

A total of 368 medicinal plant species were investigated and documented together with their medicinal uses by the Maonans, most of which were obtained from the wild ecosystems. The plants were used to treat 95 human diseases. Grinding was a widely used method to prepare traditional herbal medicines. There were significant relationships between gender and age, and between gender and informants’ knowledge of medicinal plant use. Deforestation for agricultural purposes was identified as the most destructive factor of medicinal plants, followed by drought and over-harvest.

**Conclusions:**

The species diversity of medicinal plants used by the Maonans in the study area was very rich. Medicinal plants played a significant role in healing various human disorders in the Maonan communities. However, the conflicts between traditional inheriting system and recent socio-economic changes (and other factors) resulted in the reduction or loss of both medicinal plants and associated indigenous knowledge. Thus, conservation efforts and policies, and innovation of inheriting system are necessary for protecting the medicinal plants and associated indigenous knowledge. Awareness is also needed to be raised among local Maonans focusing on sustainable utilization and management of both medicinal plants and traditional knowledge.

## Background

Traditional medicine is used to maintain people’s health, as well as to prevent, diagnose, improve or treat physical and mental illnesses all over the world [1,2]. Medicinal plants are believed to be with healing powers, and people have used them for many centuries. Aimed to modern drug discovery, traditional medicinal plants have been studied and developed which is followed the ethnobotanical lead of indigenous cures used by traditional medical systems [3-5]. Traditional medicinal knowledge, especially using medicinal plants in the developing countries, has been in existence and use, and has been a part of therapeutic practices [6]. Therefore, the investigation of plants and their uses (especially medicinal purposes) is one of the most primary human concerns and has been practiced in the world [7-12].

The traditional use of medicinal plants in China is widely accepted. The population of 55 minorities is 11.2 millions occupying 8% of China’s population, and these minorities distribute in 65% of the country’s territory. Each minority has its own medicinal characteristic, and has various experiences of medicinal knowledge [13]. Traditional medicinal plants play an important role of protecting people’s lives and health in minority regions, especially in remote and poor area [14,15]. Because of unique natural conditions and customs in the ethnic minority areas, long-term practices of using medicinal plants have formed various systems of treating diseases [16-18]. For example, Tibetan medicine is famous for treating digestive disorders, rheumatic diseases and wounds [19,20]. The Mongolians have a long history of horse riding, and their medicine is effective to deal with bone fracture and brain concussion. Yao medicine has special advantages in cancers and skin problems [21].

North Guangxi has been recognized as a rich biodiversity and world-famous karst area. With the elevation between 700–1500 m, it is obviously affected by plateau terrain and subtropical monsoon climate. Thus, the temperature difference of four seasons is small but the vertical climate changes significantly. There are more than 10 ethnic groups living in north Guangxi and formed colorful ethnic characteristic. As one of the indigenous minorities, Maonan is mainly living in Huanjiang Maonan Autonomous County, Guangxi Zhuang Autonomous Region, southwest China. The exceptional altitudinal range, topography and climatic variability in this region have fostered a center of plant species endemism. Here the majority of Maonan people rely on medicinal plants for self-medication. The Maonan medicine has made a great contribution to protect the health of local people. This is due to free access to medicinal herbs, cultural traditions and high cost of hospital treatments in the town nearby. Local people widely utilize endemic species, and they have developed their own traditional medicinal knowledge. Without writing language, Maonan people pass on their indigenous knowledge from generation to generation orally. Nowadays, the Maonan children spend most of their time in schools, where they are taught in Han language. This decreases their chances to learn about the uses of the medicinal plants from the old people. Therefore, important information about medicinal plants is easily lost in the transfer process of indigenous knowledge. With the impact of increasing modern health facilities and modern civilization in Maonan area, indigenous knowledge is depleting rapidly. Although a number of ethnobotanical documentations about several ethnic groups have been published during the past decades in China, few field ethnobotanical studies have been conducted in Maonan society. It is therefore necessary to carry out a survey to document the medicinal plants and associated indigenous knowledge in Maonan region.

Thus, the purposes of the present work were as follows: (i) to document and analyze the knowledge and use of medicinal plants by Maonan people at the study area; (ii) to circulate the results within the scientific community in order to open a door for research in other disciplines; (iii) to document the medicinal plants that could be valuable in future’s phytochemical and pharmacological discoveries, and (iv) to contribute to the knowledge and conservational possibilities of plant biodiversity, bearing in mind that biological diversity is also related to the use and applications of natural resources.

## Materials and methods

### Study area and the people

**Figure 1:**
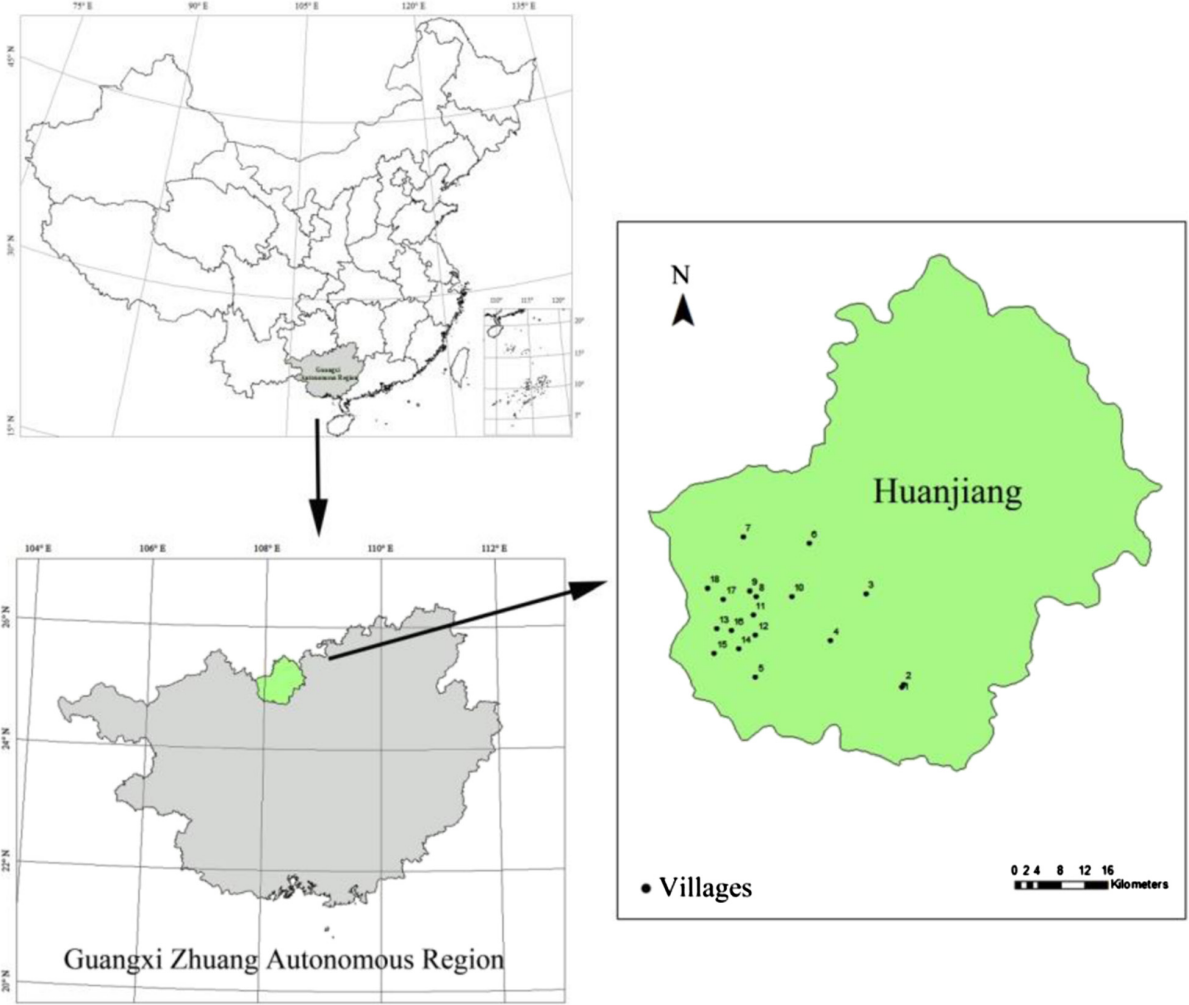
Sketch map of the study area.

The study area covered 18 villages of Huanjiang County (the only Maonan autonomous County in China) in the northern part of Guangxi Zhuang Autonomous Region, southwest China (Figure 1). The villages are located in 5 townships, which were selected based on Maonan traditional settlements, namely: Chengnan, Chengbei, Luoyang, Shuiyuan, Shangnan, Youdong, Mulun, Xia’nan, Pochuan, Fengyi, Zhongnan, Tangba, Xiatang, Yuhuan, Caimen, Guzhou, Xiyuan, and Jingyang villages. Huanjiang County is situated in the subtropical zone, located between 24°83′ and 25°06′ east longitude and between 107°92′ and 108°26′ north latitude, with the annual average temperature of 20°C and annual rain fall of 1500 mm. The vegetation of the county belongs to the subtropical evergreen montane forest. It is humid in summer and relatively dry in winter. The most Maonan villages are seated on the small strips of flat land or slopes in the rocky mountainous area at 500–1000 meters above sea level. The sinkholes and underground caverns in the area have well developed because of karst landform. Despite abundant rainfall, there are no big rivers but only a small number of streams. Water shortage has been a major obstacle to economic and social development in the Maonan areas.

The Maonan minority, with a total population of about 107,200, is one of the 55 officially recognized ethnic groups in China. With no written language [22], the Maonans’ stories and traditions are remembered and passed down orally from generation to generation, but these are becoming less and less. The Maonan language belongs to the Dong-Shui branch of the Zhuang-Dong language group in the Chinese-Tibetan language family. The Maonan language is widely spoken in Maonan communities. Almost all of the Maonans know both Han and Zhuang languages, because they need to communicate with the Zhuang and Han people, the majorities in Guangxi. About 60% of the Maonan people live in Huanjiang County, which is the only Maonan autonomous county in China. The Maonans are polytheistic, and they pay homage to dozens of deities or immortals on various occasions. These icons include figures from myths, legends, celebrities of historical events, divinities from Taoism or Buddhism, ancestors of the family and so on [22]. Due to remote mountainous regions and poor economic environment, traditional remedies of medicinal plants are the most important and sometimes the only source of therapeutics in the Maonan villages. The long utilization history and traditional knowledge of medicinal plants had supported their livelihoods. The Maonan healers and farmers have developed their own ethnomedicinal knowledge.

### Field works and ethnobotanical data collection

A total of 118 (106 males and 12 females) informants were interviewed in the study area, in which 80 were selected using snowball technique and 38 key informants were selected purposively and systematically based on the recommendations of knowledgeable elders, local authorities and development agents. All of the informants were local inhabitants aged between 21 and 85 years. Local Maonan healers were surely identified as key informants, because they were important custodians and participants of indigenous knowledge of medicinal plants. Interestingly, all these traditional healers were males. A few women were also interviewed to examine their medicinal knowledge and opinions.

Ethnobotanical investigations were carried out to collect data on medicinal plants used to treat human ailments following standard methods in Maonan area. The methodological approaches were semi-structured interviews, field observations, group discussions and guided field walks. The data were collected from June 2012 to September 2014. Interviews and discussions were undertaken based on a checklist of questions prepared in Chinese and translated into Maonan language. Information was carefully recorded during interviews with each informant. Field observations were performed with traditional healers guided on the morphological features and habitats of each medicinal plant species. Voucher specimens of cited medicinal plants were collected and their local identity was re-confirmed by other informants. The information obtained was cross-checked with the other informants. The information such as the local name, habit, wild/cultivated, availability of medicinal plants, need of conservation and efforts made by inhabitants, and traditional medicinal uses of plants were recorded. Group discussions were conducted on multipurpose, conservation, threats of the medicinal plants, and transferability of knowledge with the healers and local people in the villages. Also, the key informants were selected for preference ranking exercise.

### Specimen collection and identification

The listed medicinal plants were collected from field and gardens, and the habits of these plants were recorded. The voucher specimens were made and deposited in the Herbarium, College of Life and Environmental Sciences, Minzu University of China, Beijing, China, for future references. The botanical identities of collected specimens were confirmed by the authors and other taxonomists at Minzu University of China. Plant names were checked with *Flora of China* and botanical websites (e.g. http://www.tropicos.org/).

### Data analysis

The data were summarized using Microsoft Office Excel sheet. Descriptive statistical methods were applied to analyze and summarize the ethnobotanical data such as frequency and percentage.

Preference ranking exercise [23,24] was conducted by 8 key informants on 7 medicinal plants used to treat traumatic injury in the study area. The highest number of medicinal plants was prescribed by informants to fight traumatic injury. The plants in this exercise were short-listed by the key informants, and then their importance to manage traumatic injury was discussed. The plants were given to the informants and were ranked based on their efficacy. Medicinal plant that was believed to be the most effective was given the highest value 7, and the one with the least effectiveness a value of 1. Rank was determined based on the total score of each species. A total rank of preference exercise was obtained by summing the number of informant given.

The reported ailments were grouped into 21 categories based on the information gathered from the interviewees. Factor of informant consensus (F_IC_) was calculated for each category to test the agreements of the informants on the reported cures for the group of diseases. The F_IC_ was calculated as follows: number of use citations in each category (N_ur_) minus the number of species used (N_t_), and divided by the numbers of use citations in each category minus one [25,26]. The formula was listed as below:

$$ {\mathrm{F}}_{\mathrm{IC}}=\left({\mathrm{N}}_{\mathrm{ur}}\hbox{-} {\mathrm{N}}_{\mathrm{t}}\right)/\left(\ {\mathrm{N}}_{\mathrm{ur}}\hbox{-} 1\right) $$

## Results

### Medicinal plants reported

The study recorded 368 medicinal plant species (see Table 1). Ethnomedicinal information for each species, including scientific name, Chinese name, local name, family name, life form, habitat, plant parts used, preparation and uses, was listed in Table 1. The species belonged to 295 genera and 115 families were used by Maonan people to treat various human ailments. Among the families that contributed more medicinal species were Asteraceae, represented by 24 species (6.52%), Fabaceae with 19 (5.16%) species, and Rosaceae with 16 (4.35%), while other 292 families contributed 309 (83.97%) species were mostly represented by 1 or 2 species (Table 2).

**Table 1:** **Inventory of Medicinal Plants Traditionally Used by Maonan People**

**No.**	**Scientific name**	**Chinese name**	**Maonan name**	**Family**	**Life form**	**Habit**	**Parts used**	**Preparation and uses**
1	*Abelmoschus sagittifolius* (Kurz) Merr.	Jianyeqiukui箭叶秋葵	--	Malvaceae	Herb	Wild	Root	Grinding, decoction; Taken orally for furuncle
2	*Abrus cantoniensis* Hance	Guangdong xiangsizi广州相思子	rouŋ^2^ra^2^təp^7^	Fabaceae	Shrub	Wild	Whole plant	Grinding, decoction; Taken orally for acute and chronic hepatitis, stomachache, rheumatism, ostealgia, traumatic injury, liver cirrhosis and common cold
3	*Abutilon indicum* (L.) Sweet	Mopancao磨盘草	ruoŋ^2^ŋaŋ³luiŋ^5^	Malvaceae	Herb	Wild	Whole plant	Boiled with meat; Taken orally soup, treating for fever due to common cold, bronchitis, epidemic parotitis and tuberculosis
4	*Acanthopanax gracilistylus* W. W. Smith.	Wujia五加	mba³tshi^2^an^2^lau^4^	Araliaceae	Shrub	Both	Root, Bark	Grinding, decoction; Taken orally for rheumatic arthritis, traumatic injury, carminative, bone fracture and pain of limbs
5	*Acanthopanax trifoliatus* (L.) Merr.	Baile白簕	mba³tshi^6^man^2^ndi^5^	Araliaceae	Shrub	Both	Stem, Root	Medicinal liquor for treating rheumatic arthritis, traumatic injury, waist and legs pain, ostealgia and sciatica; Pound fresh part applied on the affected area, treating for eczema, ulcer and furuncle
6	*Achillea wilsoniana* Heimerl ex Hand. -Mazz.	Yunnanshi云南蓍	--	Asteraceae	Herb	Wild	Whole plant	Pound fresh part applied on the affected area, treating for ulcer
7	*Achyranthes bidentata* Blume	Niuxi牛膝	ma^6^wei^5^gou^2^ɣou¹	Amaranthaceae	Herb	Wild	Root	Grinding and drink with wine for traumatic injury, removing blood stasis
8	*Aconitum carmichaeli* Debx.	Wutou乌头	taŋ^5^gou²ʔno²	Ranunculaceae	Herb	Both	Tuber	Grinding, decoction; Taken orally for scrofula, perineum ache
9	*Acorus calamus* L.	Shuichangpu水菖蒲	baːŋ^5^sjɛm^2^rəm³	Acoraceae	Herb	Wild	Root	Powder; Taken orally for diarrhea
10	*Acorus tatarinowii* Schott	Shichangpu石菖蒲	ruoŋ^2^jɛŋ³vu^2^	Acoraceae	Herb	Wild	Rhizome	Grinding, decoction; Taken orally for epilepsy and convulsion
11	*Adenophora tetraphylla* (Thunb.) Fisch.	Lunyeshashen轮叶沙参	mua²ʨiɔ³gʔai²	Campanulaceae	Herb	Wild	Root	Grinding, decoction; Taken orally for complications after measles
12	*Adiantum capillus-junonis* Rupr.	tuanyutiexianjue团羽铁线蕨	ya^2^bou³	Adiantaceae	Herb	Wild	Whole plant, Rhizome	Boiled with meat and drunk the soup, treating for piles
13	*Aeginetia indica* L.	Yegu野菰	--	Orobanchaceae	Herb	Wild	Whole plant	Grinding, decoction; Taken orally for swelling, clearing away heat and toxic materials
14	*Ageratum conyzoides* L.	Huoxiangji藿香蓟	--	Asteraceae	Herb	Wild	Whole plant	Grinding, decoction; Taken orally for fever due to common cold, empyrosis and abscess
15	*Agrimonia pilosa* Ledeb.	Longyacao龙芽草	ruoŋ^2^hiu¹cia³	Rosaceae	Herb	Wild	Root	Boiled with meat or wine and drunk the soup, treating for piles, enteritis, diarrhea, hemafecia, hematuria
16	*Ainsliaea bonatii* Beauverd	Xinyetu'erfeng心叶兔儿风	ma^6^ka^6^ʑai^2^	Asteraceae	Herb	Wild	Whole plant	Grinding, decoction; Taken orally for cough, asthma with throat itching
17	*Akebia quinata* (Houtt.) Decne.	Mutong木通	--	Lardizabalaceae	Liana	Wild	Stem, Root, Fruit	Grinding, decoction; Taken orally for rheumatism, diuresis, promoting lactation
18	*Alangium chinense* (Lour.) Harms	Bajiaofeng八角枫	mei^4^da^2^	Alangiaceae	Tree	Both	Fibrous root	Grinding, decoction; Taken orally for rheumatic arthritis, lumbar muscle degeneration, asthma and bleeding
19	*Allium fistulosum* L.	Cong葱	soŋ³xien³nien^2^	Liliaceae	Herb	Homegarden	Whole plant	Grinding, decoction; Taken orally for common cold, pains, rheumatic headache, numbness of limbs and replenishing the liver
20	*Allium sativum* L.	Suan蒜	kɔŋ¹do^2^	Liliaceae	Herb	Homegarden	Bulb	Grinding, dispersed in water and drunk for pertussis cough, enteritis, tuberculosis, poor appetite, indigestion, diarrhea
21	*Allium tuberosum* Rottl. ex Spreng.	Jiu韭	mba³kən^5^	Liliaceae	Herb	Homegarden	Whole plant	Grinding, decoction; Taken orally for toothache, piles, traumatic injury and insect bite
22	*Alocasia macrorrhizos* (L.) G. Don	Reyahaiyu热亚海芋	--	Araceae	Herb	Wild	Whole plant	Pound fresh part applied on the affected area, treating for snake bite and innominate inflanunatory of unknown origin
23	*Alpinia katsumadai* Hayata	Caodoukou草豆蔻	--	Zingiberaceae	Herb	Homegarden	Fruit	Grinding, decoction; Taken orally for indigestion
24	*Alpinia oxyphylla* Miq.	Yizhi益智	--	Zingiberaceae	Herb	Wild	Fruit	Grinding, decoction; Taken orally for warming the spleen, kidney deficiency, diarrhea and spermatorrhea
25	*Alternanthera sessilis* (L.) DC.	Lianzicao莲子草	--	Amaranthaceae	Herb	Wild	Whole plant	Grinding, decoction; Taken orally for reducing fever and causing diuresis
26	*Amomum tsaoko* Crevost et Lemarie	Caoguo草果	--	Zingiberaceae	Herb	Homegarden	Fruit	Grinding, decoction; Taken orally for eliminating phlegm, indigestion, diarrhea and malaria
27	*Amomum villosum* Lour.	Sharen砂仁	--	Zingiberaceae	Herb	Homegarden	Fruit	Grinding, decoction; Taken orally for indigestion
28	*Andrographis paniculata* (Burm. f.) Nees	Chuanxinlian穿心莲	--	Acanthaceae	Herb	Wild	Whole plant	Grinding, decoction; Taken orally for clearing away heat and toxic materials
29	*Androsace umbellata* (Lour.) Merr.	Diandimei点地梅	--	Primulaceae	Herb	Wild	Whole plant	Grinding, decoction; Taken orally for inflammation and traumatic injury
30	*Anemone hupehensis* Lem.	Dapowanhuahua打破碗花花	ma^2^miŋ^5^yɛ^5^	Ranunculaceae	Herb	Wild	Root, Whole plant	Grinding, decoction; Taken orally for biliary tract ascariasis
31	*Aralia chinensis* L.	Songmu楤木	--	Araliaceae	Tree	Wild	Seed	Grinding, decoction; Taken orally for snake bite
32	*Aralia undulata* Hand.-Mazz.	Boyuansongmu波缘楤木	mei^5^ȵun^4^	Araliaceae	Shrub	Wild	Root	Boiled with meat and drunk the soup, treating for cough
33	*Arctium lappa* L.	Niubang牛蒡	maː^6^kaː^6^wei^5^	Asteraceae	Herb	Homegarden	Fruit	Grinding, decoction; Taken orally for infantile fever and cough
34	*Ardisia gigantifolia* Stapf	Zoumatai走马胎	ruoŋ^2^loŋ^2^mia^4^	Myrsinaceae	Shrub	Wild	Rhizome, Whole plant	Medicinal liquor for treating rheumatism, rheumatic arthritis, waist and legs pain, paralysis, hemiplegia and traumatic injury
35	*Ardisia japonica* (Thunb.) Blume	Zijinniu紫金牛	wa^5^ʨiɛm²wei³	Myrsinaceae	Shrub	Wild	Whole plant	Grinding, decoction; Taken orally for jaundiced hepatitis, cough, traumatic injury and preventing phlegm
36	*Arisaema erubescens* (Wall.) Schott	Yibasannanxing一把伞南星	ma¹gəp^8^tai^5^	Araceae	Herb	Wild	Tuber	Pound fresh part applied on the affected area, treating for snake bite
37	*Arisaema heterophyllum* Blume	Tiannanxing天南星	jɛk^7^khω^6^dɔŋ^2^	Araceae	Herb	Wild	Tuber	Grinding, decoction; Taken orally for traumatic injury, cough, hypertension, acute inflammation and abdomen pain
38	*Arisaema rhizomatum* C. E. C. Fischer	Xuelijian雪里见	kɣou²ŋau^4^	Araceae	Herb	Wild	Tuber	Medicinal liquor for treating scrofula and perineum ache
39	*Aristolochia fangchi* Y. C. Wu ex L. D. Chow et S. M. Hwang	Guangfangji广防己	ruoŋ^2^dak^8^loŋ²	Aristolochiaceae	Liana	Wild	Tuber	Grinding, decoction; Taken orally for acute nephritis, urinary tract infection, hypertension, rheumatic heart disease, edema
40	*Aristolochia kwangsiensis* Chun et How ex C. F. Liang	Guangximadouling广西马兜铃	--	Aristolochiaceae	Liana	Wild	Tuber	Grinding, decoction; Taken orally for snake bite, stomachache, diarrhea, strep throat, epidemic parotitis, lymphnoditis
41	*Aristolochia versicolor* S. M. Hwang	Biansemadouling变色马兜铃	--	Aristolochiaceae	Liana	Wild	Whole plant	Grinding, decoction; Taken orally for snake bite
42	*Armeniaca vulgaris* Lam.	Xing杏	dəŋ¹vɔŋ^5^ʑa^2^	Rosaceae	Tree	Homegarden	Seed	Grinding, decoction; Taken orally for chronic trachitis, cough
43	*Artemisia annua* L.	Huanghuahao黄花蒿	ruoŋ^2^nŋai^6^min³	Asteraceae	Herb	Wild	Whole plant	Grinding, decoction; Taken orally for malaria, fever, indigestion, tuberculosis hot flashes and night sweat; washing for scab, pruritus and mosquito bite
44	*Artemisia capillaris* Thunb.	Yinchenhao茵陈蒿	ma^6^ʔai³	Asteraceae	Herb	Wild	Whole plant	Grinding, decoction; Taken orally for hepatitis and jaundice
45	*Artemisia japonica* Thunb.	Muhao牡蒿	--	Asteraceae	Herb	Wild	Whole plant	Grinding, decoction; Taken orally for clearing away heat and toxic materials, inflammation and blood stasis
46	*Asarum longerhizomatosum* C. F. Liang et C. S. Yang	Xijingjin'erhuan长茎金耳环	--	Aristolochiaceae	Herb	Wild	Whole plant	Grinding, decoction; Taken orally for headache, toothache, cough, diarrhea, acute enteritis
47	*Asarum sieboldii* Miq.	Xixin细辛	ruoŋ^2^ndeŋ^5^kha³	Aristolochiaceae	Herb	Wild	Whole plant, Root	Grinding, decoction; Taken orally for cough, relieving pain
48	*Asparagus cochinchinensis* (Lour.) Merr.	Tianmendong天门冬	lak^5^mən^2^tuŋ¹	Asparagaceae	Herb	Wild	Tuber	Grinding, decoction; Taken orally for tuberculosis, cough, constipation, diabetes and sore throat after rash
49	*Azolla imbricata* (Roxb.) Nakai	Manjianghong满江红	--	Azollaceae	Herb	Wild	Whole plant	Pound fresh part applied on the affected area, treating for measles
50	*Baphicacanthus cusia* (Nees) Bremek.	Banlan板蓝	ruoŋ^2^wom¹	Acanthaceae	Herb	Homegarden	Whole plant	Grinding, decoction drunk for common cold, sore throat, parotitis and epidemic cerebrospinal meningitis
51	*Bauhinia brachycarpa* Wall.	Anyeyangtijia鞍叶羊蹄甲	yaŋm³gəm³duo^5^	Fabaceae	Tree	Both	Root, Leaf	Boiled with meat and drunk the soup, treating for cough, hemoptysis
52	*Bauhinia championii* (Benth.) Benth.	Longxuteng龙须藤	bjeu³in^5^	Fabaceae	Liana	Both	Stem	Grinding, decoction drunk for rheumatism, traumatic injury, stomachache, waist and legs pain
53	*Belamcanda chinensis* (L.) Redoute	Shegan射干	mei^5^van³biɛn²	Iridaceae	Herb	Both	Root	Grinding, decoction drunk for sore throat
54	*Berberis julianae* Schneid	Haozhuci豪猪刺	taːŋm^2^mɛn^5^sem^5^	Berberidaceae	Shrub	Wild	Root	Grinding, boiled with water and washed the affected area for clearing away heat and toxic materials, inflammation
55	*Bidens pilosa* L.	Guizhencao鬼针草	wɔk^7^cut^7^na^5^	Asteraceae	Herb	Wild	Whole plant	Grinding, decoction; Taken orally for nephritis, jaundice, rheumatism, ostealgia, diarrhea, throat ache, kidney deficiency and waist pain
56	*Bischofia javanica* Bl.	Qiufeng秋枫	--	Euphorbiaceae	Tree	Both	Stem, Leaf	Grinding, decoction; Taken orallyfor removing blood stasis, carminative, improving indigestion
57	*Bletilla striata* (Thunb. ex A. Murray) Rchb. f.	Baiji白及	kɔŋ¹nat^7^	Orchidaceae	Herb	Both	Bulb	Powder swallowed for tuberculosis and empyrosis
58	*Blumea balsamifera* (L.) DC.	Ainaxiang艾纳香	ruoŋ^2^nŋai^6^lau^4^	Asteraceae	Herb	Wild	Whole plant	Grinding, decoction; Taken orally for common cold, rheumatic arthritis, traumatic injury, dysmenorrhea and afterpains
59	*Boehmeria nivea(L.) Gaudich.*	Zhuma苎麻	mba³ŋan³	Urticaceae	Shrub	Wild	Root Bark, Leaf	Grinding, decoction; Taken orally for miscarriage prevention, hematuria, traumatic injury, bone fracture, diuresis, measles, joint sprain
60	*Bombax malabaricum* DC.	Mumian木棉	wai^5^mei^4^	Bombacaceae	Tree	Both	Flower, Root bark, Root	Grinding, decoction; Taken orally; Flower is treating for enteritis, stomach ulcer; Root bark is treating for rheumatism, traumatic injury; Root is treating for chronic nephritis gastricism, stomach ulcer, tuberculosis of cervical lymph nodes
61	*Botrychium ternatum* (Thunb.) Sw.	Yindijue阴地蕨	do^5^gʔom^2^daːŋ³	Botrychiaceae	Herb	Wild	Whole plant	Grinding, decoction; Taken orally for cough
62	*Brucea javanica* (L.) Merr.	Yadanzi鸦胆子	--	Simaroubaceae	Shrub	Wild	Seed	Grinding, decoction; Taken orally for diarrhea, malaria and chromic diarrhea
63	*Bryophyllum pinnatum* (L. f.) Oken	Luoyeshenggen落地生根	ruoŋ^2^ra^2^pu³	Crassulaceae	Herb	Wild	Whole plant	Pound fresh part applied on the affected area, treating for detumescence by detoxification, promoting blood circulation to arrest pain, draw out pus and toxin
64	*Buddleja officinalis* Maxim.	Mimenghua密蒙花	wa³kuŋ³ruo²	Loganiaceae	Shrub	Wild	Flower	Grinding, decoction; Taken orally for swelling and pain of eye, hyperdacryosis and cloudness of cornea
65	*Caesalpinia sappan* Linn.	Sumu苏木	mei^4^sam³mɔk^8^	Fabaceae	Tree	Wild	Heartwood	Grinding, decoction; Taken orally for traumatic injury, rheumatism, ostealgia, bleeding
66	*Caesalpinia sepiaria* Roxb.	Yunshi云实	ʔŋən^5^ʔniao^2^	Fabaceae	Tree	Wild	Root, Seed	Medicinal liquor for treating contraception in the menstrual period
67	*Callicarpa macrophylla* Vahl	Dayezizhu大叶紫珠	ruoŋ^2^lak^8^phau^5^	Verbenaceae	Shrub	Wild	Root, Leaf	Grinding, decoction; Taken orally for hemafecia and hemoptysis
68	*Campanumoea javanica* Bl.	Jianqianbao金钱豹	bieu³thωp^8^jou¹	Campanulaceae	Herb	Wild	Root	Powdered and swallowed for tuberculosis, enteritis, diarrhea, appendicitis, traumatic injury and piles
69	*Camptotheca acuminata* Decne.	Xishu喜树	--	Nyssaceae	Tree	Both	Fruit, Root	Grinding, decoction; Taken orally for cancer and schistosome
70	*Canscora lucidissima* (Levl. et Vaniot) Hand.-Mazz	Chuanxincao穿心草	ma^6^chuan^2^	Gentianaceae	Herb	Wild	Whole plant	Grinding, decoction; Taken orally for stranguria, snake bite, stomachache, cough and jaundiced hepatitis
71	*Capsella bursa-pastoris* (L.) Medik.	Ji荠	mba³kɔŋ¹pia³	Cruciferae	Herb	Wild	Whole plant	Grinding, decoction; Taken orally for catching common cold, fever, nephritis, edema, hypertension, enteritis
72	*Cassia tora* Linn.	Jueming决明	thou^6^maŋ³xiзŋ³	Fabaceae	Herb	Both	Seed	Grinding, decoction; Taken orally for hyperlipidemia, hepatitis, stomachache, acute conjunctivitis, habitual constipation, dental ulcer
73	*Cassytha filiformis* L.	Wugenteng无根藤	bieu³chim^6^cieu¹	Lauraceae	Herb	Wild	Stem	Grinding, decoction; Taken orally for vitiligo, jaundice, constipation, waist and knees pain, impotence and spermatorrhea
74	*Cayratia japonica* (Thunb.) Gagnep.	Wulianmei乌蔹莓	--	Vitaceae	Herb	Wild	Whole plant, Root	Medicinal liquor for paralysis
75	*Celosia argentea* L.	Qingxiang青葙	mba³pωm³pa^5^	Amaranthaceae	Herb	Wild	Whole plant	Grinding, decoction; Taken orally for trachitis, gastricism
76	*Cerastium glomeratum* Thuill.	Qiuxujuan'er球序卷耳	maː^6^ʔan^4^ʑau^2^	Caryophyllaceae	Herb	Wild	Whole plant	Pound fresh part applied on the affected area, treating for febrile convulsion
77	*Chaenomeles sinensis* (Thouin) Koehne	Mugua木瓜	--	Rosaceae	Shrub	Homegarden	Fruit	Grinding, decoction; Taken orally for smooth the liver and stomach
78	*Chirita eburnea* Hance	Niu'erduo牛耳朵	ma^5^ba^5^	Gesneriaceae	Herb	Wild	Whole plant	Grinding, decoction; Taken orally for bronchitis
79	*Chloranthus holostegius* (Handel-Mazzetti) Pei & Shan	Quanyuanjinlilan全缘金栗兰	tei³kuai^5^wa^5^	Chloranthaceae	Herb	Wild	Root	Boiled with meat and drunk the soup, treating for weakness
80	*Cinnamomum camphora* (L.) Presl	Xiangzhang香樟	mei^4^kau¹	Lauraceae	Tree	Homegarden	Bark	Grinding, decoction; Taken orally for acute gastroenteritis, rheumatism, ostealgia, emesis, diarrhea and bone fracture
81	*Cinnamomum cassia* Presl	Rougui肉桂	--	Lauraceae	Tree	Homegarden	Stem	Grinding, decoction; Taken orally for cough, dysmenorrhea and sweating
82	*Cinnamomum subavenium* Miq.	Xianggui香桂	--	Lauraceae	Tree	Both	Bark	Grinding, decoction; Taken orally for antiseptic
83	*Cirsium japonicum* Fisch. ex DC.	Daji大蓟	mba³tin³tsuok^7^lau^4^	Asteraceae	Herb	Wild	Root, Whole plant	Grinding, decoction; Taken orally for jaundice, scabies, hemafecia, muscle swelling and gastroduodenal ulcer
84	*Clematis chinensis* Osbeck.	Weilingxian威灵仙	ruoŋ^2^pek^7^mi^6^saŋ³	Ranunculaceae	Liana	Wild	Root, Leaf	Grinding, decoction; Taken orally for tonsillitis, jaundice, migraine and rheumatism
85	*Clerodendrum chinense* (Osbeck) Mabb.	Choumoli臭茉莉	ruoŋ^2^phuŋ^6^hi^6^	Verbenaceae	Shrub	Wild	Whole plant	Pound fresh part applied on the affected area, treating for rheumatic arthritis, traumatic injury, rheumatism and detumescence
86	*Clerodendrum cyrtophyllum* Turcz.	Daqing大青	--	Verbenaceae	Shrub	Wild	Leaf	Ground, decoction; Taken orally for fever due to common cold, tonsillitis, pharyngitis, parotitis, enteritis and diarrhea
87	*Coix lacryma-jobi* L.	Yiyi薏苡	ɣhou^6^gaŋ^5^yə^4^	Gramineae	Herb	Both	Root	Grinding, decoction; Taken orally for acute nephritis
88	*Colocasia antiquorum* Schott	Yeyu野芋	phi²niəŋ^6^	Araceae	Herb	Wild	Tuber	Pound fresh part applied on the affected area, treating for bleeding, furuncle, empyrosis and snake bite
89	*Commelina communis* L.	Yazhicao鸭趾草	mba³ciap^7^	Commelinaceae	Herb	Wild	Whole plant	Grinding, decoction; Taken orally for diarrhea, influenza, acute tonsillitis, edema, enteritis, urinary tract infection, empyrosis and bleeding
90	*Crataegus pinnatifida* Bunge	Shanzha山楂	dɛŋ¹miɛ^5^²yə^2^	Rosaceae	Tree	Homegarden	Fruit	Grinding, decoction; Taken orally for poor appetite, blood stasis
91	*Croton tiglium* L.	Badou巴豆	ruoŋ^2^mei^4^miət^7^	Euphorbiaceae	Tree	Homegarden	Root Bark, Leaf	Pound fresh leaf applied on the affected area, treating for bleeding, herpes zoster; Pound root bark applied on the affected area, treating for snake bite
92	*Cucumis sativus* L.	Huanggua黄瓜	--	Cucurbitaceae	Herb	Homegarden	Fruit	Pound fresh part applied on the affected area, treating for bleeding and skin whitening
93	*Curculigo orchioides* Gaertn.	Xianmao仙茅	ruoŋ^2^saŋ³thɔk^8^	Amaryllidaceae	Herb	Homegarden	Whole plant	Grinding, decoction; Taken orally for headache due to common cold, rheumatic arthritis, neurasthenia, chronic nephritis, erectile dysfunction and seminal leakage
94	*Curcuma aromatica* Salisb.	Yujin郁金	--	Zingiberaceae	Herb	Wild	Tuber	Grinding, decoction; Taken orally for bleeding, jaundice and cooling blood
95	*Curcuma longa* L.	Jianghuang姜黄	ruoŋ^2^cɛŋ³woŋ²	Zingiberaceae	Herb	Homegarden	Rhizome	Grinding, decoction; Taken orally for abnormal menstruation, amenorrhea, flatulence and blood stasis
96	*Curcuma zedoaria* (Christm.) Roscoe	E'zhu莪术	pi^6^cɛŋ³nəm³	Zingiberaceae	Herb	Homegarden	Rhizome	Grinding, decoction; Taken orally for rheumatism, ostealgia, traumatic injury, abdomen pain
97	*Cuscuta chinensis* Lam.	Tusizi菟丝子	--	Convolvulaceae	Herb	Wild	Whole plant	Grinding, decoction; Taken orally for hepatitis
98	*Cyclea hypoglauca* (Schauer) Diels	Fenyelunhuanteng粉叶轮环藤	--	Menispermaceae	Liana	Wild	Root	Grinding, decoction; Taken orally for toothache, urinary tract infection, rheumatism, diphtheria, ostealgia; Pound fresh part applied on the affected area, treating for carbuncle, snake bite
99	*Cynanchum atratum* Bunge	Baiwei白薇	lau^2^ʨiŋ^5^xi^5^	Asclepiadaceae	Herb	Wild	Root	Medicinal liquor for treating rheumatic arthritis
100	*Cynanchum auriculatum* Royle ex Wight	Niupixiao牛皮消	gʔɛ^2^lin^5^xiao^5^	Asclepiadaceae	Shrub	Wild	Root	Boiled with meat and drunk the soup, treating for infantile dry-sickness and malnutrition
101	*Cynanchum officinale* (Hemsl.) Tsiang & H.D.Zhang	Zhushateng朱砂藤	--	Asclepiadaceae	Shrub	Wild	Root	Grinding, decoction; Taken orally for pain killer and weakness
102	*Cynanchum paniculatum* (Bunge) Kitag.	Xuchangqing徐长卿	ta^6^ʔnu^2^	Asclepiadaceae	Herb	Both	Root, Whole plant	Grinding, decoction; Taken orally for enteritis and diarrhea
103	*Cyperus rotundus* L.	Xiangfuzi香附子	lak^8^rut^8^	Cyperaceae	Herb	Wild	Tuber	Grinding, decoction; Taken orally for clearing and activating the channels and collaterals, common cold, abnormal menstruation
104	*Cyrtomium fortunei* J. Sm.	Guanzhong贯众	rin³tsiɛk^7^lau^4^	Dryopteridaceae	--	Wild	Whole plant	Grinding, decoction; Taken orally for common cold, parotitis, gastrorrhagia, hematuria, postpartum lochiorrhea and body deficiency disease
105	*Datura metel* L.	Yangjinhua洋金花	--	Solanaceae	Herb	Wild	Flower	Pound and applied on the affected area for ulcer and pains
106	*Datura stramonium* L.	Mantuoluo曼陀罗	ruoŋ^2^chou^6^dun³	Solanaceae	Herb	Wild	Leaf	Pound fresh part applied on the affected area, treating for furuncle and traumatic injury
107	*Davallia mariesii* T. Moore ex Baker	Gusuibu骨碎补	xiŋ^5^bɔa^5^	Davalliaceae	--	Wild	Rhizome	Grinding, decoction; Taken orally for bone fracture and fructus psoraleae
108	*Desmodium heterocarpon* (L.) DC.	Jiadidou假地豆	thou^6^ti^5^pa^5^	Fabaceae	Shrub	Wild	Whole plant	Grinding, decoction; Taken orally for preventing mumps, epidemic encephalitis B, kidney and vesical stone
109	*Dichondra repens* J.R. Forst. & G. Forst.	Matijin马蹄金	ruoŋ²tin³mia^4^	Convolvulaceae	Herb	Wild	Whole plant	Pound fresh part applied on the affected area, treating for bleeding, urinary stone and jaundiced hepatitis
110	*Dicliptera chinensis* (L.) Juss.	Gougancai狗肝菜	ruoŋ^2^təp^7^ma³	Acanthaceae	Herb	Wild	Whole plant	Grinding, decoction; Taken orally for fever due to common cold, epidemic hepatitis B, rheumatic arthritis, conjunctivitis, diuresis and measles
111	*Dimocarpus longan* Lour.	Longyan龙眼	ruoŋ^2^kuei^4^juon²	Sapindaceae	Tree	Homegarden	Aril	Medicinal liquor for cosmetic, insomnia, forgetfulness, replenishing heart, tonic and blood deficiency
112	*Dioscorea bulbifera* L.	Huangdu黄独	lak^8^phuo^2^	Dioscoreaceae	Liana	Homegarden	Tuber	Grinding, decoction; Taken orally for cough, hemoptysis and epistaxis
113	*Dioscorea cirrhosa* Lour.	Shuliang薯莨	daŋ^5^gʔui^5^pɛ²	Dioscoreaceae	Liana	Homegarden	Tuber	Grinding, decoction; Taken orally for gastroduodenal ulcer
114	*Dioscorea opposita* Thunb.	Shuyu薯蓣	--	Dioscoreaceae	Liana	Homegarden	Tuber	Grinding, decoction; Taken orally for weakness, cough and frequent urination
115	*Diospyros kaki* Thunb.	Shi柿	den³mian^5^	Ebenaceae	Tree	Homegarden	Fruit, Persistent calyx	Pound fresh part mixing the rice wine applied on the affected area, treating for lymphadenectasis
116	*Dipsacus asperoides* C.Y. Cheng & Ai	Chuanxuduan川续断	noŋ²bu²yɛ^5^	Dipsacaceae	Herb	Wild	Seed, Root	Boiled with meat and drunk the soup, treating for leucorrhoea and bone fracture
117	*Disporum cantoniense* (Lour.) Merr.	Wanshouzhu万寿竹	ma^6^mei^5^vɛn³	Liliaceae	Herb	Wild	Root, Rhizome	Boiled with meat and drunk the soup, treating for cough
118	*Drynaria propinqua* (Wall. ex Mett.) J. Sm.	Shilianjianghujue石莲姜槲蕨	--	Drynariaceae	--	Wild	Rhizome	Medicinal liquor for treating rheumatic arthritis, traumatic injury, bone fracture and blood stasis
119	*Duchesnea indica* (Andrews) Teschem.	Shemei蛇莓	taːŋm²bei²zeŋ¹	Rosaceae	Herb	Wild	Whole plant	Pound fresh part applied on the affected area, treating for empyrosis, snake bite, furuncle
120	*Dysosma versipellis* (Hance) M. Cheng ex T.S. Ying	Bajiaolian八角莲	va^5^piat^7^lim^6^	Berberidaceae	Herb	Both	Rhizome	Grinding, decoction; Taken orally for mumps, traumatic injury, lymphnoditis, snake bite, breast carcinoma
121	*Eclipta prostrata* (L.) L.	Lichang鳢肠	wɔk^7^mək^8^	Asteraceae	Herb	Wild	Whole plant	Grinding, decoction; Taken orally for infantile diarrhea, enteritis, hemafecia, hematuria, hemoptysis and bleeding
122	*Elephantopus scaber* L.	Didancao地胆草	ruoŋ²təp^7^do^6^	Asteraceae	Herb	Wild	Whole plant	Grinding, decoction; Taken orally for common cold, acute tonsillitis, acute jaundiced hepatitis, ascites due to cirrhosis, chronic gastricism and furuncle
123	*Eleusine indica* (L.) Gaertn.	Niujincao牛筋草	ruoŋ²su^5^chin^6^	Gramineae	Herb	Wild	Whole plant	Grinding, decoction; Taken orally for traumatic injury, rheumatism, ostealgia, infantile indigestion
124	*Emilia sonchifolia* (L.) DC.	Yidianhong一点红	mba³kha³tu^5^	Asteraceae	Herb	Wild	Whole plant	Grinding, decoction; Taken orally for inflammation, sore throat, cough, fever due to common cold, urticaria, herpes zoster
125	*Epimedium brevicornu* Maximowicz Trudy Imp. S.-Peterburgsk.	Yinyanghuo淫羊藿	ma^5^gan²duo³	Berberidaceae	Herb	Both	Stem, Leaf	Medicinal liquor for treating rheumatism, tonic
126	*Epimeredi indica* (L.) Rothm.	Guangfangfen广防风	ruoŋ²woŋ²	Lamiaceae	Herb	Wild	Whole plant	Grinding, decoction; Taken orally for common cold, acute gastroenteritis; boiled with water and washed the affected area for snake bite, furuncle, eczema
127	*Equisetum arvense* L.	Wenjing问荆	gɔŋ²dau^5^	Equisetaceae	--	Wild	Whole plant	Powder swallowed for headache
128	*Equisetum hyemale* L.	Bitongcao笔筒草	--	Equisetaceae	--	Wild	Whole plant	Grinding, decoction; Taken orally for bleeding, diuresis
129	*Eriobotrya japonica* (Thunb.) Lindl.	Pipa枇杷	va³bi²ba^5^	Rosaceae	Tree	Homegarden	Leaf	Grinding, decoction; Taken orally for pertussis cough
130	*Eucalyptus robusta* Sm.	An桉	mei^4^cau^5^xui^4^	Myrtaceae	Tree	Homegarden	Leaf	Grinding, decoction; Taken orally for influenza, diarrhea
131	*Eucommia ulmoides* Oliv.	Duzhong杜仲	thu^6^tsuŋ^5^	Eucommiaceae	Tree	Both	Bark	Grinding, decoction; Taken orally for hypertension, kidney deficiency, lumbago
132	*Eupatorium chinense* L.	Duoxugong多须公	--	Asteraceae	Herb	Wild	Root	Grinding, decoction; Taken orally for clearing away heat and toxic materials, blood stasis, traumatic injury
133	*Euphorbia antiquorum* L.	Huoyangle火殃勒	ruoŋ²ko³loŋ²	Euphorbiaceae	Shrub	Wild	Whole plant	Pound fresh part and fried with wine, applied on the affected area, treating for furuncle, innominate inflanunatory of unknown origin
134	*Euphorbia chrysocoma* H. Lév. & Vaniot	Shuihuanghua水黄花	maː³nom²ʔan²	Euphorbiaceae	Herb	Wild	Root	Grinding, decoction; Taken orally for infectious hepatitis
135	*Euphorbia hirta* L.	Feiyangcao飞扬草	ruoŋ²jɛŋ³thuŋ^6^thin^6^	Euphorbiaceae	Herb	Wild	Whole plant	Grinding, decoction; Taken orally for bacillary diarrhea, enteritis, bronchitis, nephritis
136	*Euphorbia humifusa* Willdenow	Dijin地锦	--	Euphorbiaceae	Herb	Wild	Whole plant	Grinding, decoction; Taken orally for malaria, diuresis
137	*Euphorbia milii* Des Moul.	Tiehaitang铁海棠	ruoŋ²ndunŋ³waŋ³	Euphorbiaceae	Shrub	Both	Whole plant	Pound fresh part applied on the affected area, treating for carbuncle
138	*Euphorbia thymifolia* L.	Qian'gencao千根草	--	Euphorbiaceae	Herb	Wild	Whole plant	Grinding, decoction; Taken orally for bacillary diarrhea, enteritis, diarrhea, piles, bleeding
139	*Evodia lepta* (Spreng.) Merr.	Sanyaku三桠苦	ruoŋ²sam³tsha³	Rutaceae	Tree	Wild	Root, Leaf	Grinding, decoction; Taken orally for epidemic meningitis, influenza, fever, epidemic encephalitis B
140	*Evodia rutaecarpa* (Juss.) Benth.	Wuzhuyu吴茱萸	tsha^6^la^6^	Rutaceae	Shrub	Wild	Fruit	Grinding, decoction; Taken orally for diarrhea, abnormal menstruation, diseases of liver stasis, emesis
141	*Fagopyrum tataricum* (L.) Gaertn.	Kuqiao苦荞	--	Polygonaceae	Herb	Wild	Fruit	Grinding, decoction; Taken orally for stomachache, indigestion
142	*Fallopia multiflora* (Thunb.) Haraldson	Heshouwu何首乌	mən^6^daŋ³yɛ^5^	Polygonaceae	Herb	Both	Tuber, Stem	Grinding, decoction; Taken orally for weakness
143	*Fibraurea recisa* Pierre	Tianxianteng天仙藤	--	Menispermaceae	Liana	Wild	Root	Grinding, decoction; Taken orally for headache, fever, acute tonsillitis, strep throat, diarrhea, jaundiced hepatitis, gastricism, enteritis
144	*Ficus microcarpa* L. f.	Rongshu榕树	ruoŋ²mei^4^joŋ²	Moraceae	Tree	Homegarden	Leaf	Grinding, decoction; Taken orally for flu, malaria, bronchitis, acute enteritis, bacillary diarrhea, pertussis cough, tonsillitis
145	*Ficus tikoua* Bureau	Diguo地果	--	Moraceae	Liana	Wild	Whole plant	Grinding, decoction; Taken orally for jaundice, diarrhea and internal injury
146	*Flemingia prostrata* Roxb.	Qianjinba千斤拔	ruoŋ²sωt^7^khui²	Fabaceae	Shrub	Wild	Root	Grinding, decoction or medicinal liquor drunk for lumbar muscle degeneration, traumatic injury, rheumatic arthritis and tonsillitis
147	*Gardenia jasminoides* J. Ellis	Zhizi栀子	lak^8^kei³	Rubiaceae	Shrub	Both	Fruit	Grinding, decoction; Taken orally for jaundiced hepatitis, fever, diarrhea, nephritis and edema
148	*Gastrodia elata* Blume	Tianma天麻	ŋoŋ^5^bu^4^noŋ²	Orchidaceae	Herb	Wild	Rhizome	Grinding, decoction; Taken orally for headache and stomachache
149	*Gaultheria leucocarpa* var. yunnanensis (Franch.) T.Z. Hsu & R.C. Fang	Baiguobaizhu白果白珠	mei^5^ʔa^5^	Ericaceae	Shrub	Wild	Whole plant, Root	Grinding, decoction; Taken orally for rheumatic arthritis and traumatic injury
150	*Gelsemium elegans* (Gardner & Champ.) Benth.	Gouwen钩吻	ruoŋ²sai³mu^5^	Loganiaceae	Liana	Wild	Whole plant	Pound fresh part applied on the affected area, treating for furuncle, carbuncle
151	*Gentiana rhodantha* Franch.	Honghualongdan红花龙胆	ya^5^ma²mənp^8^	Gentianaceae	Herb	Wild	Root	Grinding, decoction; Taken orally for furuncle
152	*Geranium nepalense* Sweet	Nibo'er'laoguancao尼泊尔老鹳草	ma^6^ʑaŋ^5^nan^5^	Geraniaceae	Herb	Wild	Whole plant	Grinding, decoction; Taken orally for pertussis cough
153	*Gerbera piloselloides* (L.) Cass.	Maodadingcao毛大丁草	ruoŋ²təp^7^thi^6^	Asteraceae	Herb	Wild	Whole plant	Grinding, decoction; Taken orally for clearing away heat and toxic materials, fever due to common cold, cough, diarrhea, infantile indigestion
154	*Geum aleppicum* Jacq.	Lubianqing路边青	mba³men²	Rosaceae	Herb	Wild	Whole plant	Boiled with meat and drunk the soup, treating for deficiency of dizziness
155	*Ginkgo biloba* L.	Yinxing银杏	--	Ginkgoaceae Engler	Tree	Homegarden	Fruit, Leaf	Grinding, decoction; Taken orally for moistening lung, cough
156	*Gleditsia sinensis* Lam.	Zaojia皂荚	--	Fabaceae	Tree	Both	Pod	Grinding, decoction; Taken orally for apocenosis, detumescence
157	*Glochidion puberum* (Linnaeus) Hutchinson	Suanpanzi算盘子	mei^4^thω^6^teŋ^5^	Euphorbiaceae	Shrub	Wild	Root, Leaf	Grinding, decoction; Taken orally for bacillary diarrhea, infantile indigestion, diarrhea, abdomen pain, proctoptosis, migraine , lymphnoditis
158	*Gomphrena globosa* L.	Qianrihong千日红	xien³vən³lan¹	Amaranthaceae	Herb	Wild	Flower	Grinding, decoction; Taken orally for asthma, bronchitis, pertussis cough, tuberculosis, diarrhea and hemoptysis
159	*Gonostegia hirta* (Blume ex Hassk.) Miq.	Nuomituan糯米团	--	Urticaceae	Herb	Wild	Whole plant, Root	Grinding, decoction or boiled with meat and drunk for clearing away heat and removing dampness, innominate inflanunatory of unknown origin
160	*Gymnotheca chinensis* Decne.	Luoshuo裸蒴	maŋ^5^wɛŋ³bɔa^5^	Saururaceae	Herb	Homegarden	Whole plant	Boiled with meat and drunk the soup, treating for weakness and cough
161	*Gynostemma pentaphyllum* (Thunb.) Makino	Jiaogulan绞股蓝	--	Cucurbitaceae	Liana	Wild	Whole plant	Grinding, decoction; Taken orally for rheumatism, bronchitis and stomachache
162	*Hedyotis diffusa* Willd.	Baihuasheshecao白花蛇舌草	ruoŋ²ma²rui²sɛ^5^	Rubiaceae	Herb	Wild	Whole plant	Grinding, decoction; Taken orally for hepatitis, cough, bronchitis, tonsillitis
163	*Hemsleya sphaerocarpa* Kuang & A. M. Lu	Shelian蛇莲	tei^5^ʔŋaːn²	Cucurbitaceae	Liana	Wild	Tuber	Powdered; Taken orally for appendicitis
164	*Homalomena occulta* (Lour.) Schott	Qiannianjian千年健	ma^6^moŋ³ʨɛ^5^	Araceae	Herb	Wild	Rhizome	Grinding, decoction; Taken orally for rheumatism, numbness of limbs, traumatic injury, bone fracture
165	*Houttuynia cordata* Thunb.	Yuxingcao鱼腥草	mba³wət^8^	Saururaceae	Herb	Homegarden	Whole plant	Grinding, decoction; Taken orally for edema, bronchopneumonia, nephritis, enteritis, diarrhea, cough
166	*Hydrocotyle nepalensis* Hook	Hongmaticao红马蹄草	--	Umbelliferae	Herb	Wild	Whole plant	Pound fresh part mixing with hot liquor and applied on the affected area, treating for traumatic injury
167	*Hydrocotyle sibthorpioides* Lam.	Tianhusui天胡荽	na^5^ʨiao²nɛm³	Umbelliferae	Herb	Wild	Whole plant	Grinding, decoction; Taken orally for headache due to common cold
168	*Hypericum japonicum* Thunb.	Tianjihuang田基黄	ruoŋ²kha³kai^5^	Guttiferae	Herb	Wild	Whole plant	Grinding, decoction; Taken orally for hepatitis, acute conjunctivitis, tonsillitis and forepart hepatocirrhosis
169	*Hypericum sampsonii* Hance	Yuanbaocao元宝草	wa³ciɛn³	Guttiferae	Herb	Wild	Whole plant	Grinding, decoction; Taken orally for traumatic injury, pain, indigestion, chest congestion
170	*Illicium difengpi* B.N. Chang	Difengpi地枫皮	--	Magnoliaceae	Shrub	Wild	Stem, Bark	Grinding, decoction; Taken orally for rheumatism, rheumatic arthralgia and lumbar muscle degeneration
171	*Impatiens balsamina* L.	Fengxianhua凤仙花	wa³dip^7^sim¹	Balsaminaceae	Herb	Homegarden	Whole plant, Seed	Pound fresh part applied on the affected area, treating for furuncle, carbuncle
172	*Impatiens pinfanensis* Hook. f.	Kuaijiefengxianhua块节凤仙花	fan^4^mɛ^5^ma²	Balsaminaceae	Herb	Homegarden	Tuber	Pound fresh part applied on the affected area, treating for scrofula
173	*Imperata cylindrica* (L.) P. Beauv.	Baimao白茅	taŋ^5^ya³guaŋ^4^	Gramineae	Herb	Wild	Rhizome	Grinding, decoction; Taken orally for nephritis, edema, bleeding
174	*Ipomoea cairica* (L.) Sweet	Wuzhaojinlong五爪金龙	ruoŋ²lak^8^oŋ^5^	Convolvulaceae	Herb	Both	Leaf, Tuber	Pound fresh part applied on the affected area, treating for carbuncle, clearing away heat and toxic materials
175	*Ipomoea mauritiana* Jacq.	Qizhualong七爪龙	miau²ren³sen^5^	Convolvulaceae	Liana	Both	Tuber, Leaf	Boiled with meat and drunk the soup, treating for nephritis
176	*Ipomoea pescaprae* (L.) R. Br.	Houteng厚藤	ruoŋ²an³mia^4^	Convolvulaceae	Herb	Wild	Whole plant	Grinding, decoction; Taken orally for rheumatic lumbocrural pain and lumbar muscle degeneration
177	*Iris tectorum* Maxim	Yuanwei鸢尾	ʑo^5^waːŋ¹	Iridaceae	Herb	Both	Rhizome	Pound fresh part with water is taken as a drink for improving indigestion
178	*Juglans regia* L.	Hutao胡桃	den³van^5^kɔŋ²	Juglandaceae	Tree	Both	Fruit	Grinding, decoction; Taken orally for tonic, back pain
179	*Juncus effusus* L.	Dengxincao灯心草	ȵan^6^daːŋ^5^	Juncaceae	Herb	Wild	Whole plant	Grinding, decoction; Taken orally for jaundiced hepatitis
180	*Justicia gendarussa* Burm. f.	Xiaobogu小驳骨	ruoŋ²tiək^7^dak^8^sɛ^5^	Acanthaceae	Shrub	Wild	Stem, Leaf	Pound fresh part applied on the affected area, treating for bone fracture, traumatic injury, 2rheumatic arthritis, ulcer
181	*Justicia ventricosa* Wall. ex Hook. f.	Heiyexiaobogu黑叶小驳骨	--	Acanthaceae	Shrub	Wild	Stem, Leaf	Pound fresh part applied on the affected area, treating for bone fracture, traumatic injury, rheumatic arthritis, waist pain, bleeding
182	*Kadsura heteroclita* (Roxb.) Craib	Yixingnanwuweizi异形南五味子	ruoŋ²li^5^rωp^8^	Magnoliaceae	Liana	Wild	Stem	Grinding, decoction or infusion with wine drunk for bone fracture, ostealgia, chronic gastricism, acute gastroenteritis
183	*Kadsura longipedunculata* Finet & Gagnep.	Nanwuweizi南五味子	--	Magnoliaceae	Liana	Wild	Fruit	Decoctionn; Taken orally for cough, insomnia
184	*Kalimeris indica* (L.) Sch. Bip.	Malan马兰	ruoŋ²xien³sɔk^7^	Asteraceae	Herb	Wild	Whole plant	Grinding, decoction; Taken orally for pneumonia, bronchitis
185	*Kyllinga brevifolia* Rottb.	Duanyeshuiwugong短叶水蜈蚣	--	Cyperaceae	Herb	Wild	Whole plant	Grinding, decoction; Taken orally for infantile malnutrition, helminth
186	*Laggera alata* (D. Don) Sch. Bip. ex Oliv.	Liulengju六棱菊	ruoŋ²jɛn³nəm¹	Asteraceae	Herb	Wild	Whole plant	Grinding, decoction; Taken orally for rheumatic arthritis, nephritis, edema
187	*Laportea cuspidata* (Wedd.) Friis	Aima艾麻	tuɔm²rɛn^5^	Urticaceae	Herb	Wild	Whole plant, Root	Medicinal liquor for rheumatic arthritis
188	*Leonurus artemisia* (Lour.) S.Y. Hu	Yimucao益母草	ra²loŋ²cit^7^vən^6^	Lamiaceae	Herb	Wild	Whole plant	Grinding, decoction; Taken orally for edema, nephritis, abnormal menstruation, promoting blood circulation due to menstruation
189	*Ligusticum chuanxiong* S.H. Qiu, Y.Q. Zeng, K.Y. Pan, Y.C. Tang & J.M. Xu	Chuanxiong川芎	ta^5^chuan²wɔŋ^5^	Umbelliferae	Herb	Both	Rhizome	Grinding, decoction; Taken orally for carminative, activate blood for acesodyne
190	*Ligusticum sinense* Oliv.	Gaoben藁本	--	Umbelliferae	Herb	Wild	Whole plant	Medicinal liquor for waist pain, kidney deficiency
191	*Ligustrum lucidum* W.T. Aiton	Nüzhen女贞	--	Oleaceae	Tree	Homegarden	Fruit	Grinding, decoction; Taken orally for tonifying kieney and liver
192	*Ligustrum robustum* subsp. chinense P.S. Green	Cuzhuangnüzhen粗壮女贞	va³zhɛ^5^gaŋm²	Oleaceae	Tree	Homegarden	Leaf	Drink like the tea for dizziness
193	*Lilium brownii* F.E. Brown ex Miellez	Yebaihe野百合	kɔŋ¹dɔ²pa^5^	Liliaceae	Herb	Both	Bulb	Grinding, decoction; Taken orally for tuberculosis, edema, insomnia, neurasthenia and palpitation
194	*Lindera aggregata* (Sims) Kosterm.	Wuyao乌药	--	Lauraceae	Shrub	Wild	Root	Grinding, decoction; Taken orally for stomachache, abdomon pain
195	*Litchi chinensis* Sonn.	Lizhi荔枝	--	Sapindaceae	Tree	Homegarden	Stone fruit	Grinding, decoction; Taken orally for rheumatism, pain and removing moping
196	*Lithospermum erythrorhizon* Siebold & Zucc.	Zicao紫草	gaŋ^5^pat^8^	Boraginaceae	Herb	Wild	Root	Grinding, decoction; Taken orally for measles
197	*Litsea cubeba* (Lour.) Pers.	Shanjijiao山鸡椒	--	Lauraceae	Shrub	Both	Fruit	Pound fresh/dry fruit, decoction; Taken orally for cough, diarrhea, stomachache, toothache, bleeding
198	*Litsea pungens* Hemsl.	Mujiangzi木姜子	ruoŋ²mei^4^saŋ¹	Lauraceae	Tree	Both	Root	Grinding, decoction; Taken orally for gastricism
199	*Livistona chinensis* (Jacq.) R. Br. ex Mart.	Pukui蒲葵	ruoŋ²xien^5^phu²	Palmae	Tree	Homegarden	Seed	Grinding, decoction; Taken orally for cancer
200	*Lobelia chinensis* Lour.	Banbianlian半边莲	nun³mua²ʔnɛm^4^	Campanulaceae	Herb	Wild	Whole plant	Grinding, boiled with water and washed the affected area for snake bite
201	*Lobelia sequinii* Levl. et Vant.	Xi'nanshan'gengcai西南山梗菜	ruoŋ²thai^6^tsɛŋ¹cuωn³	Campanulaceae	Herb	Wild	Whole plant	Grinding, boiled with water and washed the affected area for rheumatic arthritis, traumatic injury, ulcer
202	*Lonicera japonica* Thunb.	Rendong忍冬	wa³cim³mən²	Caprifoliaceae	Liana	Both	Stem, Flower	Grinding and decoction; Taken orally; Stem is for jaundice, clearing away heat and toxic materials, headache and fever; flower is for enteritis, diarrhea, pneumonia, influenza
203	*Lophatherum gracile* Brongn.	Danzhuye淡竹叶	mei^4^tim¹sɛ^5^	Gramineae	Herb	Both	Leaf	Grinding, decoction; Taken orally for urinary tract infection, aphthous stomatitis, swelling, aching of gum
204	*Loropetalum chinense* (R. Br.) Oliv.	Jimu檵木	ruoŋ²mei^4^ci^5^	Hamamelidaceae	Shrub	Wild	Leaf, Flower, Root	Grinding, decoction; Taken orally; Leaf is for abdomen pain, metrorrhagia; Flower is for bleeding; Root is for traumatic injury, chronic arthritis, amenorrhea, bleeding
205	*Lycopodium japonicum* Thunb.	Shisong石松	mʔau²muan^4^	Lycopodiaceae	--	Wild	Whole plant	Grinding, decoction; Taken orally for rheumatic arthritis, arthralgia, leg cramp, hand and foot numbness
206	*Lycopus lucidus* Turcz. ex Benth.	Disun地笋	--	Lamiaceae	Herb	Wild	Whole plant	Grinding, decoction; Taken orally for abnormal menstruation, amenorrhea, traumatic injury, bone fracture
207	*Lygodium japonicum* (Thunb.) Sw.	Haijinsha海金沙	ma²goŋ²bou³	Lygodiaceae	--	Wild	Whole plant, Sporangium	Pound fresh part applied on the affected area, treating for chronic ulcer, skin infection, furuncle, foot rot
208	*Lysimachia christinae* Hance	Guoluhuang过路黄	ma^6^gʔou²ʔan²	Primulaceae	Herb	Wild	Whole plant	Grinding, decoction; Taken orally for urinary tract infection, jaundice, hepatitis
209	*Lysimachia paridiformis* Franch.	Luodimei落地梅	--	Primulaceae	Herb	Wild	Whole plant	Grinding, decoction; Taken orally for infantile convulsions
210	*Lysionotus pauciflorus* Maxim.	Diaoshijutai吊石苣苔	ba^5^dau³ma^4^	Gesneriaceae	Shrub	Wild	Whole plant	Grinding, decoction; Taken orally for bronchitis, asthma
211	*Magnolia officinalis* Rehder & E.H. Wilson	Houpo厚朴	--	Magnoliaceae	Tree	Both	Bark	Grinding, decoction; Taken orally for emesis, diarrhea
212	*Mahonia bealei* (Fortune) Carrière	Kuoyeshidagonglao阔叶十大功劳	ruoŋ²waŋ^6^lien^4^	Berberidaceae	Shrub	Wild	Root	Grinding, decoction; Taken orally for pneumonia, tuberculosis, infectious hepatitis, acute gastroenteritis, bronchitis
213	*Mallotus apelta* (Lour.) Müll. Arg.	Baibeiye白背叶	mei^4^phiau^6^sei¹	Euphorbiaceae	Shrub	Wild	Root, Leaf	Grinding, decoction; Taken orally; Root is for chronic hepatitis, hepatosplenomegaly, gestational edema, enteritis, diarrhea; Leaf is for traumatic injury, otitis media, furuncle, bleeding, thrush
214	*Mallotus barbatus* Müll. Arg.	Maotong毛桐	--	Euphorbiaceae	Shrub	Wild	Leaf	Pound fresh part and applied on the affected area, treating for clearing away heat and toxic materials, bed ulcer, eczema
215	*Marsilea quadrifolia* L.	Ping苹	phuŋ^6^phieu²lau^4^	Marsileaceae	--	Wild	Whole plant	Grinding, decoction; Taken orally for jaundiced hepatitis, asthma, edema, hepatic ascites, fever due to common cold
216	*Melastoma candidum* D. Don	Yemudan野牡丹	ruoŋ²lak^8^ma^5^ndi^5^	Melastomataceae	Shrub	Wild	Whole plant	Grinding, decoction; Taken orally for hemafecia, watery diarrhea
217	*Melastoma dodecandrum* Lour.	Dinie地菍	lak^8^nin¹	Melastomataceae	Shrub	Wild	Whole plant	Grinding, decoction; Taken orally for removing blood stasis, traumatic injury, diarrhea, lithangiuria, urinary obstruction
218	*Melia azedarach* L.	Lian楝	ruoŋ²ku¹lien^4^	Meliaceae	Tree	Both	Fruit, Leaf	Grinding, boiled with water and washed the affected area for scabies, tinea capitis and rice paddies dermatitis
219	*Mimosa pudica* L.	Hanxiucao含羞草	ruoŋ²ra²nŋei³	Fabaceae	Herb	Both	Whole plant	Grinding, decoction; Taken orally for insomnia
220	*Mirabilis jalapa* L.	Zimoli紫茉莉	ruoŋ²jɛn³wa³	Nyctaginaceae	Herb	Both	Root	Boiled with meat and drunk the soup, treating for leucorrhoea, abnormal menstruation, prostatitis, metrorrhagia
221	*Momordica cochinchinensis* (Lour.) Spreng.	Mubiezi木鳖子	tiŋ^5^ndiŋ^5^ka³	Cucurbitaceae	Liana	Wild	Seed, Leaf, Stem	Grinding, decoction; Taken orally for innominate inflanunatory of unknown origin, carbuncle, lymphnoditis
222	*Morus alba* L.	Sang桑	ruoŋ²tshaŋ¹	Moraceae	Tree	Both	Leaf, Bark	Grinding, decoction; Taken orally for lung heat panting and cough, hypertension, edema
223	*Munronia henryi* Harms	Aituotuo矮陀陀	--	Meliaceae	Shrub	Wild	Whole plant	Medicinal liquor for traumatic injury
224	*Murraya exotica* L.	Jiulixiang九里香	ruoŋ²mei^4^ndaŋ³	Rutaceae	Tree	Both	Root, Leaf	Grinding, decoction; Taken orally for rheumatism, ostealgia, traumatic injury, toothache and stomachache
225	*Mussaenda pubescens* W.T. Aiton	Yuyejinhua玉叶金花	ruoŋ²phiɛ³va^5^phuok^8^	Rubiaceae	Shrub	Both	Stem, Leaf	Grinding, decoction; Taken orally for hyperthermia, influenza, tonsillitis, enteritis, diarrhea and sphagitis
226	*Myrica rubra* (Lour.) Siebold & Zucc.	Yangmei杨梅	lak^8^se^5^	Myricaceae	Tree	Homegarden	Root Bark	Grinding, decoction; Taken orally for traumatic injury, bone fracture, diarrhea, stomach and duodenal ulcer
227	*Nandina domestica* Thunb.	Nantianzhu南天竹	waŋ^6^liɛn^4^sɛ^5^	Berberidaceae	Shrub	Wild	Root, Stem, Fruit	Grinding, decoction; Taken orally; Root and stem are for cough, fever, conjunctivitis, diarrhea, jaundice, hepatitis, traumatic injury. Fruit is for cough, asthma, pertussis
228	*Nepeta cataria* L.	Jingjie荆芥	--	Lamiaceae	Herb	Homegarden	Whole plant	Grinding, decoction; Taken orally for common cold
229	*Nephrolepis cordifolia* (L.) C. Presl	Shenjue肾蕨	lak^8^ȵən^4^	Davalliaceae	--	Wild	Rhizome, Leaf, Whole plant	Grinding, decoction; Taken orally for fever due to common cold, cough, diarrhea, acute enteritis, jaundiced hepatitis
230	*Oenanthe javanica* (Blume) DC.	Shuiqin水芹	maː^6^ʨip^7^ʑam^5^	Umbelliferae	Herb	Wild	Whole plant	Grinding, decoction; Taken orally for hypertension
231	*Ophioglossum reticulatum* L.	Xinyeping'erxiaocao心叶瓶尔小草	ruoŋ²ma²rui²	Ophioglossaceae	--	Wild	Whole plant	Pound fresh part applied on the affected area, treating for furuncle, snake bite and acute conjunctivitis
232	*Ophiopogon japonicus* (L. f.) Ker Gawl.	Maidong麦冬	ruoŋ²lak^8^ju³	Liliaceae	Herb	Both	Tuber	Grinding, decoction; Taken orally for chronic bronchitis, cough
233	*Opuntia stricta* (Haw.) Haw. var. *dillenii* (Ker-Gawl.) Benson	Xianrenzhang仙人掌	ma²mωm^4^	Cactaceae	Shrub	Both	Stem	Pound fresh part applied on the affected area, treating for parotitis, carbuncle, empyrosis
234	*Oroxylum indicum* (L. ) Kurz	Muhudie木蝴蝶	mei^4^ən³eu^5^	Bignoniaceae	Tree	Homegarden	Bark, Seed	Grinding, decoction; Taken orally for acute bronchitis, tuberculosis, jaundiced hepatitis, sore throat
235	*Osbeckia opipara* C.Y. Wu & C. Chen	Chaotianguan朝天罐	--	Melastomataceae	Shrub	Wild	Root	Boiled with meat and drunk the soup, treating for tonic, bleeding, diarrhea
236	*Oxalis corniculata* L.	Cujiangcao酢浆草	mba³thωm^6^sou¹	Oxalidaceae	Herb	Wild	Whole plant	Pound fresh part applied on the affected area, treating for febrile convulsion, enteritis, diarrhea, parotitis
237	*Paederia scandens* (Lour.) Merr.	Jishiteng鸡矢藤	bieu³tωt^7^ma³	Rubiaceae	Liana	Wild	Whole plant, Root	Medicinal liquor for treating flu, cough, pertussis cough, diarrhea, stomachache, chest stuffiness
238	*Paeonia lactiflora* Pall.	Shaoyao芍药	--	Ranunculaceae	Herb	Homegarden	Root	Powder tied for pain and blood stasis
239	*Paeonia suffruticosa* Andrew	Mudan牡丹	ma^5^muan^4^	Ranunculaceae	Shrub	Homegarden	Root Bark	Grinding, decoction; Taken orally for lobar pneumonia
240	*Palhinhaea cernua* (L.) Vasc. & Franco	Chuisuishisong垂穗石松	--	Lycopodiaceae	--	Wild	Whole plant	Grinding, decoction; Taken orally for relaxing tendons and activating collaterals, carminative, blood stasis, bleeding
241	*Paris polyphylla* Sm.	Qiyeyizhihua七叶一枝花	wa^6^ten^5^va¹	Trilliaceae	Herb	Wild	Whole plant	Pound fresh part applied on the affected area, treating for traumatic injury and snake bite
242	*Pentasacme championii* Benth.	Shiluomo石萝藦	ruoŋ²saŋ³nut^8^	Asclepiadaceae	Herb	Wild	Whole plant	Grinding, decoction; Taken orally for rheumatism, ostealgia, traumatic injury and ascites due to cirrhosis; Pound fresh part applied on the affected area, treating for snake bite, herpes zosters
243	*Perilla frutescens* (L.) Britton	Zisu紫苏	mba³ha^5^lan¹	Lamiaceae	Herb	Homegarden	Whole plant	Grinding, decoction; Taken orally for common cold, cough, asthma, emesis
244	*Periploca forrestii* Schltr.	Heilonggu黑龙骨	mei^5^ʑa²nam^5^	Asclepiadaceae	Shrub	Wild	Whole plant	Medicinal liquor for treating rheumatic arthritis
245	*Phellodendron amurense* Rupr.	Huangbo黄檗	mei^5^bɛ²ʔan³	Rutaceae	Tree	Wild	Bark	Grinding, decoction; Taken orally for diabetes insipidus
246	*Pholidota chinensis* Lindl.	Shixiantao石仙桃	ruoŋ²xien³thui²	Orchidaceae	Herb	Wild	Whole plant	Grinding, decoction; Taken orally for cough, tuberculosis, scrofula, diuresis, infantile malnutrition
247	*Phragmites australis* (Cav.) Trin. ex Steud.	Luwei芦苇	gaŋ^5^diɛ²nau^5^	Gramineae	Herb	Wild	Root	Grinding, decoction; Taken orally for infantile whitish aphthae
248	*Phyllanthus urinaria* L.	Yexiazhu叶下珠	thuŋ^6^thin^6^sei¹	Euphorbiaceae	Herb	Wild	Whole plant	Grinding, decoction; Taken orally for jaundiced hepatitis, diarrhea, enteritis, nephritis, edema and lithangiuria
249	*Phyllodium pulchellum* (L.) Desv.	Paiqianshu排钱树	ruoŋ²vak^8^rjen²	Fabaceae	Shrub	Wild	Leaf, Root	Grinding, decoction; Taken orally for fever, diarrhea, malaria, hepatitis, rheumatic ostealgia, traumatic injury, schistosome
250	*Physalis angulata* L.	Kuzhi苦蘵	--	Solanaceae	Herb	Wild	Whole plant	Grinding, decoction; Taken orally for epidemic parotitis, cough, jaundice, hepatitis, diarrhea
251	*Phytolacca acinosa* Roxb.	Shanglu商陆	lak^8^phək^8^doŋ²	Phytolaccaceae	Herb	Both	Root	Grinding, decoction; Taken orally for cervical erosion, digestibility ulcer, liver ascites, constipation, diuresis
252	*Pilea cavaleriei* H. Lév.	Shiyoucai石油菜	bma³ju²thui²	Urticaceae	Herb	Wild	Whole plant	Grinding, decoction; Taken orally for lung heat panting, cough, tuberculosis, traumatic injury, empyrosis, furuncle
253	*Piper hancei* Maxim.	Shanju山蒟	tshuon^5^pi^6^fuŋ¹	Piperaceae	Liana	Wild	Stem, Leaf	Grinding, decoction; Taken orally for lumbar muscle degeneration, chronic gastricism, cough, ostealgia, rheumatic arthritis, heatstroke, numbness of limbs
254	*Pistia stratiotes* Linnaeus Sp.	Dapiao大漂	--	Araceae	Herb	Wild	Whole plant	Pound fresh part applied on the affected area, treating for removing blood stasis
255	*Plantago asiatica* L.	Cheqian车前	mba³bɔk^8^	Plantaginaceae	Herb	Wild	Whole plant	Grinding, decoction; Taken orally for urinary tract infection, urinary stone, fever and cough due to common cold, nephritis, edema, bronchitis, hypertension
256	*Platycodon grandiflorus* (Jacq.) A. DC.	Jiegeng桔梗	--	Campanulaceae	Herb	Both	Root	Grinding, decoction; Taken orally for inflammation, cough
257	*Plumbago zeylanica* L.	Baihuadan白花丹	ruoŋ²ra²vɔk^7^	Plumbaginaceae	Herb	Wild	Whole plant	Grinding, decoction; Taken orally for traumatic injury
258	*Pogonia japonica* Rchb. f.	Zhulan朱兰	ma^6^ʑa^4^zao²	Orchidaceae	Herb	Wild	Whole plant	Boiled with meat and drunk the soup, treating for enuresis
259	*Polygala japonica* Houtt.	Guazijin瓜子金	ya¹yiŋ^4^ʑɛm²	Polygalaceae	Herb	Wild	Whole plant	Grinding, decoction; Taken orally for neurasthenia
260	*Polygonatum cyrtonema* Hua	Duohuahuangjing多花黄精	xiŋ²ʑa²	Liliaceae	Herb	Wild	Rhizome	Pound fresh part mixed with rice wine, applied on the affected area, treating for lymphadenectasis
261	*Polygonatum odoratum* (Mill.) Druce	Yuzhu玉竹	--	Liliaceae	Herb	Homegarden	Rhizome	Grinding, decoction; Taken orally for moistening lung for rresting cough
262	*Polygonatum sibiricum* Redouté	Huangjing黄精	ruoŋ²siŋ³mωmŋ^4^	Liliaceae	Herb	Both	Rhizome	Grinding, decoction; Taken orally for tuberculosis, diabetes, hypertension, weakness after ill, invigorating spleen, reinforcing stomach
263	*Polygonum aviculare* L.	Shegan射干	laŋ^5^lu^5^kun²	Polygonaceae	Herb	Wild	Whole plant	Grinding, decoction; Taken orally for stranguria due to hematuria
264	*Polygonum chinense* L.	Huotanmu火炭母	va^5^mba³sωm¹	Polygonaceae	Herb	Wild	Whole plant, Rhizome	Grinding, decoction; Taken orally for diarrhea, enteritis, indigestion, hepatitis, pharyngitis. Pound fresh part applied on the affected area, treating for traumatic injury, furuncle, eczema, dermatitis, pruritus
265	*Polygonum hydropiper* L.	Shuiliao辣蓼	mba³we^5^	Polygonaceae	Herb	Both	Whole plant	Grinding, decoction; Taken orally for diarrhea, acute ulcer, common cold, typhoid, rheumatism, ostealgia, traumatic injury. Pound fresh part applied on the affected area, treating for eczema, centipede bite
266	*Polygonum perfoliatum* (L.) L.	gangban'gui杠板归	ruoŋ²tin³diək^8^	Polygonaceae	Herb	Wild	Whole plant	Grinding, decoction; Taken orally for jaundice, diarrhea, malaria, nephritis, edema. Pound fresh part applied on the affected area, treating for furuncle, eczema, carbuncle
267	*Portulaca oleracea* L.	Machixian马齿苋	ruoŋ²mba³nəm¹	Portulacaceae	Herb	Wild	Whole plant	Grinding, decoction; Taken orally for acute cystitis, diarrhea, hypertension
268	*Potentilla chinensis* Ser.	Weilingcai委陵菜	ma^6^ʔgou²dui³	Rosaceae	Herb	Wild	Whole plant	Grinding, decoction; Taken orally for clearing away heat and toxic materials, diarrhea
269	*Potentilla reyniana* Bornm.	Sanyeweilingcai三叶委陵菜	--	Rosaceae	Herb	Wild	Root	Grinding, decoction; Taken orally for preventing rabies
270	*Potentilla kleiniana* Wight & Arn.	Shehanweilingcai蛇含委陵菜	ɣo^6^bei^6^rɛnm^4^	Rosaceae	Herb	Wild	Whole plant	Grinding the fresh part, decoction drunk for infantile fever
271	*Prunella vulgaris* L.	Xiakucao夏枯草	--	Lamiaceae	Herb	Wild	Whole plant	Grinding, decoction; Taken orally for clearing away heat and toxic materials
272	*Psoralea corylifolia* L.	Buguzhi补骨脂	--	Fabaceae	Herb	Wild	Seed	Medicinal liquor for treating rheumatism and kidney deficiency
273	*Pteris multifida* Poir.	Jinglanbiancao井栏边草	ruoŋ²sωt^7^kai^5^	Pteridaceae	--	Wild	Whole plant	Grinding, decoction; Taken orally for diarrhea, jaundiced hepatitis, hemafecia, hematuria
274	*Pueraria lobata* (Willd.) Ohwi	Ge葛	bieu³chai^5^	Fabaceae	Liana	Wild	Tuber	Grinding, decoction; Taken orally for fever, hypertension, protecting the liver, promoting salivation
275	*Pulsatilla chinensis* (Bunge) Regel	Baitouweng白头翁	wɔk^7^fian³puok^8^	Ranunculaceae	Herb	Wild	Rhizome	Grinding, decoction; Taken orally for diarrhea, malaria, dysmenorrhea, uterine bleeding
276	*Punica granatum* L.	Shiliu石榴	lak^8^liu²	Punicaceae	Shrub	Homegarden	Pericarp	Grinding, decoction; Taken orally for diarrhea, acute enteritis, piles, proctoptosis
277	*Pyrola calliantha* Andres	Luticao鹿蹄草	--	Pyrolaceae	Herb	Wild	Whole plant	Grinding, decoction; Taken orally for cough, weakness
278	*Pyrrosia lingua* (Thunb.) Farw.	Shiwei石韦	mba³mei^4^ri²	Polypodiaceae	--	Wild	Whole plant	Grinding, decoction; Taken orally for senile chronic bronchitis, pneumonia, nephritis, edema, urinary tract infection
279	*Quisqualis indica* L.	Shijunzi使君子	lak^6^rəm²	Combretaceae	Liana	Wild	Seed	Chewed for infantile malnutrition product, depriving ascarid
280	*Rabdosia ternifolia* (D. Don) H. Hara	Niuweicao牛尾草	tɛ^5^vɛn³ŋoŋ²	Lamiaceae	Herb	Wild	Whole plant, Leaf	Grinding, decoction; Taken orally for hepatitis, enteritis, common cold
281	*Raphanus sativus* L.	Luobo萝卜	vɛ³loŋ^5^bu³	Cruciferae	Herb	Homegarden	Seed	Grinding, decoction; Taken orally for senile chronic bronchitis
282	*Rauvolfia verticillata* (Lour.) Baill.	Luofumu萝芙木	--	Apocynaceae	Shrub	Both	Root	Pound fresh part applied on the affected area, treating for bleeding, pain killer, hypertension, dispersing blood stasis
283	*Rehmannia glutinosa* (Gaertn.) Libosch. ex Fisch. & C.A. Mey.	Dihuang地黄	ma^6^liao²lip^7^	Scrophulariaceae	Herb	Wild	Tuber	Grinding, decoction; Taken orally for removing heat to promote salivation
284	*Reineckia carnea* (Andr.) Kunth.	Jixiangcao吉祥草	taŋ^6^kəp^8^	Liliaceae	Herb	Wild	Whole plant	Grinding, decoction; Taken orally for bronchitis
285	*Reynoutria japonica* Houtt.	Huzhang虎杖	ruoŋ²waŋ^6^chin^6^	Polygonaceae	Herb	Wild	Rhizome	Grinding, decoction; Taken orally for cough, blood stasis, rheumatism, traumatic injury, jaundice, amenorrhea
286	*Rhoeo discolor* (L'Hér.) Hance ex Walp.	Zibeiwannianqing紫背万年青	ruoŋ²phuoŋ²wa³	Commelinaceae	Herb	Wild	Flower	Grinding, decoction; Taken orally for cough, pertussis cough, diarrhea, hemoptysis, sore throat, scrofula
287	*Rhus chinensis* Mill.	Yanfumu盐麸木	mei^4^wωt^7^	Anacardiaceae	Shrub	Both	Cecidium	Grinding, decoction; Taken orally for bleeding, arrest sweating, piles, pharyngitis, inflammation
288	*Ricinus communis* L.	Bima蓖麻	thuŋ^6^ju^6^	Euphorbiaceae	Herb	Homegarden	Seed	Pound fresh part applied on the affected area, treating for scabies
289	*Rorippa indica* (L.) Hiern	Hancai蔊菜	ma²you^5^yɛ^5^	Cruciferae	Herb	Wild	Whole plant	Pound fresh part and mixed with rapeseed oil, applied on the affected area, treating for dermatitis
290	*Rosa chinensis* Jacq.	Yuejihua月季花	ŋɛŋ^4^ŋɛŋ^4^ʑən^5^	Rosaceae	Shrub	Homegarden	Flower	Grinding, decoction; Taken orally for abnormal menstruation
291	*Rosa laevigata* Michx.	Jinyingzi金樱子	lak^8^man^4^	Rosaceae	Shrub	Both	Root, Fruit	Grinding, decoction; Taken orally for bone fracture, traumatic injury, appendicitis, diarrhea, enteritis, stomachache
292	*Rosa multiflora* Thunb.	Yeqiangwei野蔷薇	--	Rosaceae	Shrub	Wild	Root, Seed	Grinding, decoction; Taken orally for clearing and activating the channels and collaterals, diuresis
293	*Rosa roxburghii* Tratt.	Saosihua缫丝花	taŋ^5^dɛnm³gaŋ^4^	Rosaceae	Shrub	Wild	Root	Grinding, decoction; Taken orally for indigestion, stomachache
294	*Rubus parvifolius* L.	Maomei茅莓	lak^8^thωm^6^pha³	Rosaceae	Herb	Wild	Whole plant	Grinding the fresh part, decoction; Taken orally for jaundice, toothache, chronic hepatitis, stomachache, diarrhea, sphagitis
295	*Rumex nepalensis* Spreng.	Nibo'er'suanmo尼泊尔酸模	maː^6^ʔan^6^lou^5^	Polygonaceae	Herb	Wild	Root	Grinding, decoction; Taken orally for nephritis
296	*Salvia miltiorrhiza* Bunge	Danshen丹参	dan^5^sen^5^	Lamiaceae	Herb	Homegarden	Root	Grinding, decoction; Taken orally for afterpains, removing blood stasis
297	*Sambucus chinensis* Lindl.	Jiegucao接骨草	--	Caprifoliaceae	Herb	Wild	Rhizome	Grinding, decoction; Taken orally for rheumatic arthritis, tonsillitis, rheumatoid arthritis, urinary tract infection
298	*Sambucus williamsii* Hance	Jiegumu接骨木	ruoŋ²ra²liem²	Caprifoliaceae	Shrub	Both	Leaf	Grinding, decoction; Taken orally for traumatic injury, rheumatic arthritis, waist and legs pain, bone fracture, scapulohumeral periarthritis
299	*Sanguisorba officinalis* L.	Diyu地榆	gaŋ^5^gu²va³	Rosaceae	Herb	Wild	Root	Grinding the fresh part, decoction; Taken orally for diarrhea
300	*Sapindus mukorossi* Gaertn.	Wuhuanzi无患子	ruoŋ²lak^8^rək^7^	Sapindaceae	Tree	Wild	Seed	Grinding, decoction; Taken orally for tuberculosis, pertussis cough
301	*Sapium discolor* (Champ. ex Benth.) Müll. Arg.	Shanwujiu山乌桕	ruoŋ²mei^4^ək^7^	Euphorbiaceae	Tree	Both	Leaf	Grinding, decoction; Taken orally for traumatic injury, snake bite, constipation, carbuncle
302	*Sargentodoxa cuneata* (Oliv.) Rehder & E.H. Wilson	Daxueteng大血藤	bieu³phiat^7^	Lardizabalaceae	Liana	Wild	Root, Stem	Medicinal liquor for treating rheumatic arthritis, traumatic injury, ostealgia
303	*Saurauia tristyla* DC.	Shuidongge水东哥	--	Actinidiaceae	Shrub	Homegarden	Root	Grinding, decoction; Taken orally for carbuncle, cough, bronchitis, toothache
304	*Saururus chinensis* (Lour.) Baill.	Sanbaicao三白草	ruoŋ²sωt^7^mbei¹	Saururaceae	Herb	Both	Whole plant	Grinding, decoction; Taken orally for nephritis, edema, lithangiuria, eczema, furuncle, carbuncle
305	*Saxifraga stolonifera* Curtis	Hu'er'cao虎耳草	ruoŋ²kha³mωm^4^	Saxifragaceae	Herb	Wild	Leaf	Pound fresh part applied on the affected area, treating for traumatic hemorrhage, furuncle, parotitis, empyrosis
306	*Schefflera heptaphylla* (L.) Frodin	E'zhangchai鹅掌柴	mei^5^dian²ʔɛp^8^	Araliaceae	Tree	Both	Root Bark, Stem Bark, Leaf	Grinding and decoction; Taken orally; Root and Stem bark are for fever, rheumatism, ostealgia, traumatic injury, sore throat; Leaf is for eczema, allergic dermatitis
307	*Schizocapsa plantaginea* Hance	Lieguoshu裂果薯	suei¹lo^6^pu^4^	Taccaceae	Herb	Wild	Rhizome	Grinding, decoction; Taken orally for cough, traumatic injury, pharyngitis, heart and stomach pain
308	*Scutellaria barbata* D. Don	Banzhilian半枝莲	ruoŋ²wɔk^7^lim^6^sɛ^5^	Lamiaceae	Herb	Wild	Whole plant	Grinding, decoction; Taken orally for cancer, appendicitis, hepatitis and hepatic ascites
309	*Selaginella moellendorffii* Hieron.	Jiangnanjuanbai江南卷柏	ʔguit^7^miɛ²bua^5^	Selaginellaceae	--	Wild	Whole plant	Pound fresh part applied on the affected area, treating for hematoma after contusion
310	*Selaginella tamariscina* (P. Beauv.) Spring	Juanbai卷柏	ruoŋ²sai³thui²	Selaginellaceae	--	Wild	Whole plant	Grinding, decoction; Taken orally for hemafecia, epistaxis, metrorrhagia, traumatic injury, chronic hepatitis, proctoptosis
311	*Semiaquilegia adoxoides* (DC.) Makino	Tiankui天葵	ma³ɣe^5^ŋɔ²	Ranunculaceae	Herb	Wild	Tuber	Grinding, decoction; Taken orally for stomachache
312	*Senecio scandens* Buch.-Ham. ex D. Don	Qianliguang千里光	wa³nuk^8^so^5^	Asteraceae	Herb	Wild	Whole plant	Grinding, decoction; Taken orally for fever, jaundiced hepatitis, throat ache, mumps, bleeding, eczema
313	*Senna occidentalis* (L.) Link	Wangjiangnan望江南	--	Fabaceae	Shrub	Both	Seed	Grinding, decoction; Taken orally for habitual constipation, hypertension, headache, indigestion, epifolliculitis, oral mucosa ulcer
314	*Serissa japonica* (Thunb.) Thunb.	Liuyuexue六月雪	taŋ^5^ʔnui^5^wai³	Rubiaceae	Shrub	Wild	Whole plant	Grinding, decoction; Taken orally for infantile convulsions
315	*Setcreasea purpurea* Boom	Zizhumei紫竹梅	--	Commelinaceae	Herb	Wild	Whole plant	Pound fresh part applied on the affected area, treating for bleeding, snake bite, activating blood and herpes
316	*Sida szechuensis* Matsuda	Badusan拔毒散	--	Malvaceae	Shrub	Wild	Whole plant	Pound fresh part applied on the affected area, treating for traumatic injury and inflammation
317	*Sigesbeckia orientalis* L.	Xixian豨莶	wɔk^7^cut^7^btio¹	Asteraceae	Herb	Wild	Whole plant	Grinding, decoction; Taken orally for insomnia, hypertension, acute jaundiced hepatitis, diarrhea, malaria, numbness of limbs
318	*Smilax glabra* Roxb.	Tufuling土茯苓	lak^8^dəm^4^sei¹	Smilacaceae	Herb	Homegarden	Rhizome	Grinding, decoction; Taken orally for nephritis, diarrhea, detoxication, arthralgia
319	*Solanum capsicoides* All.	Niuqiezi牛茄子	--	Solanaceae	Herb	Wild	Whole plant	Grinding, decoction; Taken orally for fever due to common cold, headache, cough, abscess, chest stuffiness
320	*Solanum violaceum* L.	Citianqie刺天茄	lak^8^khat^8^se^5^	Solanaceae	Shrub	Wild	Leaf, Fruit	Pound fresh part applied on the affected area, treating for yellow-water ulcer, fingers ulcer and ringworm
321	*Solidago decurrens* Lour.	Yizhihuanghua一枝黄花	wɔk^7^wa³man¹	Asteraceae	Herb	Wild	Whole plant	Grinding, decoction; Taken orally for fever, headache, jaundice, bronchitis, acute gastricism, upper respiratory infection, swelling, throat ache
322	*Sophora flavescens* Aiton	Kushen苦参	ruoŋ²ŋau³in^5^	Fabaceae	Herb	Wild	Rhizome	Grinding, decoction; Taken orally for piles, cutaneous pruritus
323	*Sophora tonkinensis* Gagnep.	Yuenanhuai越南槐	--	Fabaceae	Shrub	Wild	Root	Grinding, decoction; Taken orally for acute pharyngitis, tonsillitis, swelling and aching of gum, cough, constipation
324	*Sparganium stoloniferum* (Buch.-Ham. ex Graebn.) Buch.-Ham. ex Juz.	Heisanleng黑三棱	--	Sparganiaceae	Herb	Wild	Tuber	Grinding, decoction; Taken orally for blood stasis, pain killer
325	*Spatholobus sinensis* Chun & T.C. Chen	Hongxueteng红血藤	ruoŋ²pu¹phiat^7^	Fabaceae	Liana	Wild	Stem	Medicinal liquor for treating traumatic injury
326	*Spatholobus suberectus* Dunn	Mihuadou密花豆	--	Fabaceae	Liana	Wild	Stem	Grinding, decoction; Taken orally for stomachache, enriching blood, waist and knees pain
327	*Spiranthes sinensis* (Pers.) Ames	Shoucao绶草	ruoŋ²thou^6^neŋ^4^	Orchidaceae	Herb	Wild	Whole plant, Root	Grinding, decoction; Taken orally for diabetes, leucorrhoea, weakness, sore throat, neurasthenia and erectile dysfunction
328	*Stahlianthus involucratus* (King ex Baker) Craib	Tutianqi土田七	ruoŋ²iŋ³doŋ²	Zingiberaceae	Herb	Both	Tuber	Grinding, decoction; Taken orally for traumatic injury, rheumatism, ostealgia
329	*Stemona tuberosa* Lour.	Dabaibu大百部	lak^8^ru³khui²	Stemonaceae	Liana	Wild	Tuber	Grinding, decoction; Taken orally for pertussis cough, tuberculosis, bronchitis
330	*Stephania cepharantha* Hayata	Jinxiandiaowugui金线吊乌龟	mɛi^5^miu²	Menispermaceae	Liana	Wild	Tuber	Pound fresh part applied on the affected area, treating for carbuncle, snake bite
331	*Streptocaulon juventas* (Lour.) Merr.	Anxiaoteng暗消藤	--	Asclepiadaceae	Liana	Wild	Root, Leaf	Grinding, decoction; Taken orally; Root is for diarrhea, piles, pneumonia, vitiligo and arrhythmia; Pound fresh leaf applied on the affected area, treating for snake bite, eczema and vaginitis
332	*Striga asiatica* (L.) Kuntze	Dujiaojin独脚金	ruoŋ²ra²mei³	Scrophulariaceae	Herb	Wild	Whole plant	Grinding, decoction; Taken orally for infantile malnutrition, dampness-heat constitution, diarrhea, jaundiced hepatitis
333	*Strophanthus divaricatus* (Lour.) Hook. & Arn.	Yangjiaoniu羊角拗	--	Apocynaceae	Shrub	Wild	Stem, Leaf	Grinding, decoction; Taken orally for rheumatic arthritis, traumatic injury, snake bite, sprain
334	*Tadehagi triquetrum* (L.) H. Ohashi	Hulucha葫芦茶	tsha²ja¹	Fabaceae	Shrub	Both	Whole plant	Grinding, decoction; Taken orally for nephritis, enteritis, diarrhea, hepatitis
335	*Talinum paniculatum* (Jacq.) Gaertn.	Turenshen土人参	kau^5^li^6^sωn¹	Portulacaceae	Herb	Wild	Root	Boiled with meat and drunk the soup, treating for moistening lung, health tonic
336	*Taraxacum mongolicum* Hand.-Mazz.	Pugongying蒲公英	mba³kat^7^sei¹	Asteraceae	Herb	Wild	Whole plant	Grinding, decoction; Taken orally for conjunctivitis, epidemic parotitis, enteritis, gastricism, hepatitis, diarrhea, acute mastitis, sphagitis
337	*Taxillus chinensis* (DC.) Danser	Guangjisheng广寄生	--	Loranthaceae	Shrub	Wild	Whole plant	Grinding, decoction; Taken orally for numbness of limbs, rheumatism, ostealgia, arthritis, lumbar muscle degeneration
338	*Tetrapanax papyrifer* (Hook.) K. Koch	Tongtuomu通脱木	tai^5^poŋ²	Araliaceae	Shrub	Homegarden	Stem pith	Boiled with meat and drunk the soup, treating for promoting lactation
339	*Tetrastigma planicaule* (Hook. f.) Gagnep.	Biandanteng扁担藤	mʔau^5^biɛn²	Vitaceae	Liana	Both	Root, Stem	Fried the root or stem, fumigation for pinkeye
340	*Tinospora sagittata* (Oliv.) Gagnep.	Qingniudan青牛胆	kɔŋ¹piɛŋ^5^vi³	Menispermaceae	Liana	Wild	Tuber	Powder, dispersed in water and drunk for acute gastroenteritis, acute pharyngitis, bacillary diarrhea, appendicitis
341	*Tinospora sinensis* (Lour.) Merr.	Zhonghuaqingniudan中华青牛胆	yuoŋ²soŋ³jin³	Menispermaceae	Liana	Wild	Stem	Grinding, decoction; Taken orally for rheumatism, traumatic injury, lumbar muscle degeneration, sciatica
342	*Toddalia asiatica* (L.) Lam.	Feilongzhangxue飞龙掌血	cim³ce³vin¹	Rutaceae	Liana	Wild	Root Bark	Pound fresh part applied on the affected area, treating for traumatic injury, skin disease, relieving pain, detumescence
343	*Trachelospermum jasminoides* (Lindl.) Lem.	Luoshi络石	--	Apocynaceae	Liana	Wild	Whole plant	Pound fresh part applied on the affected area, treating for bleeding, rheumatism, waist pain, dispersing blood stasis
344	*Trachycarpus fortunei* (Hook.) H. Wendl.	Zonglü棕榈	wei^5^	Palmae	Tree	Homegarden	Leaf, Fruit	Boiled with meat and drunk the soup, treating for epilepsy
345	*Trichosanthes kirilowii* Maxim.	Gualou栝楼	--	Cucurbitaceae	Liana	Wild	Root	Grinding, decoction; Taken orally for removing heat to promote salivation, expel pus and disperse swelling
346	*Trichosanthes rosthornii* Harms	Zhonghuagualou中华栝楼	gua^5^ʔe^5^ma²	Cucurbitaceae	Liana	Wild	Shuck, Seed	Grinding, decoction; Taken orally for edema
347	*Typhonium blumei* Nicolson & Sivad.	Litoujian犁头尖	lak^8^chieu^4^dɔŋ²	Araceae	Herb	Wild	Tuber	Pound fresh part applied on the affected area, treating for snake bite, scrofula, traumatic injury, hemangioma and furuncle
348	*Typhonium giganteum* Engl.	Dujiaolian独角莲	--	Araceae	Herb	Both	Tuber	Grinding, decoction; Taken orally for gastroduodenal ulcer
349	*Uncaria rhynchophylla* (Miq.) Miq. ex Havil.	Gouteng钩藤	mei^5^gʔau²dau³	Rubiaceae	Liana	Wild	Hooked stem	Grinding, decoction; Taken orally for jaundiced hepatitis, dizziness, headach, calming the liver
350	*Urena lobata* L.	Ditaohua地桃花	ruoŋ²wɔk^7^cut^7^	Malvaceae	Herb	Wild	Whole plant	Grinding, decoction; Taken orally for fever, diarrhea, enteritis, malaria; Pound fresh part applied on the affected area, treating for traumatic injury, bone fracture, snake bite, mastitis
351	*Valeriana jatamansi* Jones	Zhizhuxiang蜘蛛香	ma²va³	Valerianaceae	Herb	Wild	Rhizome	Pound fresh part applied on the affected area, treating for furuncle
352	*Ventilago leiocarpa* Benth.	Yiheguo翼核果	--	Rhamnaceae	Shrub	Wild	Root	Pound fresh part applied on the affected area, treating for traumatic injury, rheumatism, numbness of limbs, edema and menorrhagia
353	*Verbena officinalis* L.	Mabiancao马鞭草	ruoŋ²pien³mia^4^	Verbenaceae	Herb	Wild	Whole plant	Grinding, decoction; Taken orally for hypertension, diarrhea, malaria, nephritis, fever due to common cold, urinary tract infection
354	*Vernonia cinerea* (L.) Less.	Yexiangniu夜香牛	ruoŋ²məm^5^ndaŋ³	Asteraceae	Herb	Wild	Whole plant	Pound fresh part applied on the affected area, treating for snake bite, swelling, furuncle
355	*Viola inconspicua* Blume	Chang'e'jincai长萼堇菜	va^5^mba³kuei³	Violaceae	Herb	Wild	Whole plant	Grinding, decoction; Taken orally for pharyngitis, jaundice, diarrhea, swelling, pain of eye
356	*Viola philippica* Cav.	Zihuadiding紫花地丁	ya^5^mɛp^8^li²	Violaceae	Herb	Wild	Whole plant	Grinding, decoction; Taken orally for appendicitis, piles
357	*Viscum liquidambaricolum* Hayata	Fengxianghujisheng枫香槲寄生	sap^7^mei^4^hu³	Loranthaceae	Shrub	Wild	Whole plant	Grinding, decoction; Taken orally for lumbar muscle degeneration, cough, traumatic injury, rheumatic arthritis
358	*Vitex negundo* L.	Huangjing黄荆	mei^4^ciŋ³	Verbenaceae	Shrub	Wild	Stem, Leaf	Grinding, decoction; Taken orally for diarrhea, malaria, enteritis, common cold, heatstroke
359	*Vitex trifolia* L.	Manjing蔓荆	mei^5^ʨiɛ²ʑa²	Verbenaceae	Shrub	Wild	Fruit	Powder, swallowed for headache
360	*Wikstroemia indica* (L.) C.A. Mey.	Liaogewang了哥王	ruoŋ²ljɛŋljeu^4^	Thymelaeaceae	Shrub	Wild	Stem, Leaf	Grinding, decoction; Taken orally for clearing away heat and toxic materials, traumatic injury, hepatitis, parotitis
361	*Woodwardia japonica* (L. f.) Sm.	Gouji狗脊	waŋ^6^cin^5^kou¹	Blechnaceae	Herb	Wild	Rhizome	Grinding, decoction; Taken orally for neurasthenia, rheumatic arthralgia, diuresis, waist and knees pain
362	*Wrightia laevis* Hook. f.	Lanshu蓝树	--	Apocynaceae	Tree	Homegarden	Root, Leaf	Grinding, decoction; Taken orally for bleeding, traumatic injury, mumps
363	*Xanthium sibiricum* Patrin ex Widder	Cang'er苍耳	wɔk^7^cut^7^lau^4^	Asteraceae	Herb	Wild	Fruit	Pound after fried and drunk with yellow wine for enteritis, rheumatic arthralgia, headache
364	*Zanthoxylum armatum* DC.	Zhuyehuajiao竹叶花椒	lak^8^xieu³na^4^	Rutaceae	Tree	Both	Fruit	Grinding, decoction; Taken orally for traumatic injury, chronic gastricism, cough, depriving ascarid
365	*Zanthoxylum nitidum* (Roxb.) DC.	Liangmianzhen两面针	lak^8^xieu³doŋ²	Rutaceae	Liana	Wild	Root, Stem, Leaf	Grinding, decoction; Taken orally for duodenal ulcer, traumatic injury, rheumatism, diarrhea, malaria, chronic gastricism
366	*Zea mays* L.	Yumi玉米	nui^5^wei^5^die³	Gramineae	Herb	Homegarden	Column	Powder swallowed for diabetes
367	*Zehneria indica* (Lour.) Keraudren	Laoshuladonggua老鼠拉冬瓜	lak^8^kua³no¹	Cucurbitaceae	Liana	Wild	Whole plant	Grinding, decoction; Taken orally for urinary tract infection, tonsillitis, acute conjunctivitis, carbuncle
368	*Ziziphus jujuba* Mill.	Zao枣	zaːo³ziː²	Rhamnaceae	Tree	Homegarden	Fruit	Grinding, decoction; Taken orally for infantile diarrhea

The distribution of informants in age, gender and education class was shown in Table 3. The majority of informants interviewed were above 40 years old in this investigation. The male informants were 89.8% and less educated. There was a significant correlation between the informant age and phytomedicinal knowledge.

**Table 2:** **Taxonomic diversity of medicinal plants in the study area**

**Family**	**Number of genera**	**Percentage (%)**	**Number of species**	**Percentage of species (%)**
Asteraceae	22	7.46	24	6.52
Fabaceae	15	5.08	19	5.16
Rosaceae	11	3.73	16	4.35
Euphorbiaceae	8	2.71	14	3.80
Liliaceae	9	3.05	13	3.53
Araceae	7	2.37	11	2.99
Lamiaceae	9	3.05	9	2.45
Polygonaceae	4	1.36	8	2.17
Zingiberaceae	4	1.36	8	2.17
Lauraceae	4	1.36	7	1.90
Ranunculaceae	6	2.03	7	1.90
Rutaceae	5	1.69	7	1.90
Asclepiadaceae	4	1.36	7	1.90
Cucurbitaceae	6	2.03	7	1.90
Gramineae	6	2.03	6	1.63
Araliaceae	4	1.36	6	1.63
Rubiaceae	6	2.03	6	1.63
Verbenaceae	4	1.36	6	1.63
Other families	162	54.92	188	51.09
Total	295	100	368	100

**Table 3:** **Demographic profile of informants**

**Indicator**	**Description**	**Frequency (%)**
Age	20-29	7 (5.9)
	30-39	23 (19.5)
	40-49	38 (32.2)
	50-59	29 (24.6)
	60-69	12 (10.2)
	70-79	5 (4.2)
	≥80	4 (3.4)
Gender	Male	106 (89.8)
	Female	12 (10.2)
Education	None	27 (22.9)
	Primary	72 (61.0)
	Secondary	13 (11.0)
	Tertiary	6 (5.1)

### Life forms, plant parts used, method of collection and administration

**Figure 2:**
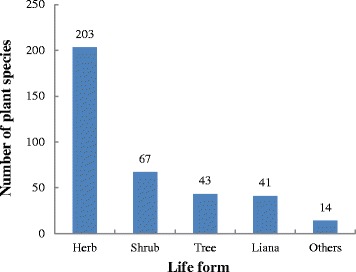
Life forms of medicinal plants in the study area.

The result of life form analysis of medicinal plants showed that herbaceous plants constituted the highest proportion represented by 203 (55.16%) species, while there were 67 (18.21%) shrubs species, 43 (11.68%) lianas and 41 (11.14%) tree species (Figure 2).

**Figure 3:**
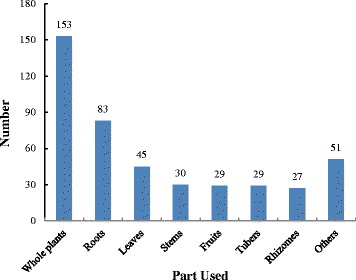
Plant parts used for the treatment of human ailments.

Informants of the study area used different plant parts for preparation of traditional drugs (e.g. leaves, roots, seeds, barks and fruits). The informants reported that more species (153) of medicinal plants were harvested for their whole plants, and these were followed by roots (83), leaves (45), stems (30), fruits (29), tubers (29), rhizomes (27) and 51 other parts (seed, bark, flower and so on) (Figure 3). The majority of remedies were prepared from fresh materials, and some were prepared from either dried or fresh materials while a few were only used from dried materials.

Of these 368 species of medicinal plants collected from the study area, most of them (256, 67.72%) were obtained from the wild habitats whereas 54 (14.67%) were from home gardens, and only 58 (15.76%) species were from both home gardens and wild habitats (see Table 1). The majority of plants used as medicine were freely harvested by healers from natural environment, while some exotic or difficult-accessed species were bought from medicinal materials suppliers. Generally fresh parts were wild harvest. Most medicinal plants were not available from local market, only some species were found to be sold but mainly for their uses as spice or food, such as *Zanthoxylum armatum*, *Nepeta cataria* and *Houttuynia cordata*.

### Diseases treated in the study area

The medicinal plants were used to treat 95 human ailments in the study area. With regard to human diseases, traumatic injury was the one against which a high number of medicinal plants (67 species) were prescribed, followed by diarrhea (65 species), cough (44 species), hepatitis (37 species), enteritis (35 species), rheumatism (30 species), arthritis (27 species), bleeding (26 species), snake bite (24 species), furuncle (22 species) and nephritis (22 species).

The highest number of species (139, 37.57%) was used for the treatment of internal organs like liver (hepatitis, cirrhosis, jaundice, hepatic ascites, hepatosplenomegaly and so on), stomach (stomachache, stomach ulcer, gastroduodenal ulcer, flatulence, gastricism, indigestion and poor appetite), enteron (enteritis, proctoptosis, appendicitis and so on), spleen and diarrhea, with 251 (20.69%) of all conditions (Table 4 Rheumatic problems (83 species used, 22.4%) were mentioned as 140 (11.54%) of all uses; 83 species (22.4%) were used to treat respiratory problems, with 112 applications (9.23%). Bone problems were treated with 72 species (19.46%), with 85 conditions (7.01%). Skin problems were mentioned in 87 uses (7.17%), with 65 species (17.57%) used for treatment. Inflammation was treated with 48 species (12.97%), and comprised 67 (5.52%) of all conditions (Table 4).

**Table 4:** **Informant consensus factor by categories of diseases in the study area**

**Category**	**Number of spp.**	**Total of spp. (%)**	**Number of use citation**	**Total of use citations (%)**	**F**_**IC**_
Stomach, intestine and liver diseases (Internal Organ)	139	37.57	236	10.69	0.41
Respiratory system	83	22.43	153	6.93	0.46
Rheumatic problems	83	22.43	217	9.83	0.62
Traumatic injury and sprain	72	19.46	275	12.46	0.74
Skin diseases, skin cut and wound	65	17.57	152	6.89	0.58
Urinary system	47	12.70	105	4.76	0.56
Inflammation	48	12.97	143	6.48	0.67
Infectious diseases	40	10.81	78	3.53	0.49
Fever and malaria	36	9.73	132	5.98	0.73
Bleeding and hemorrhages	36	9.73	95	4.30	0.63
Pain	30	8.11	64	2.90	0.54
Animal bite (snake, centipede, mosquito and bat)	30	8.11	86	3.90	0.66
Gynecological problems	29	7.84	54	2. 45	0.47
Infantile diseases	28	7.57	110	4.98	0.75
Heart and circulatory system	25	6.76	42	1.90	0.41
Male problems	25	6.76	76	3.44	0.68
Nerves and psychosomatic problems	12	3.24	14	0.63	0.15
Hyperlipidemia and diabetes	6	1.62	13	0.59	0.58
Brain diseases	5	1.35	7	0.32	0.33
Cancer and tumors	4	1.08	6	0.27	0.40
Other Uses (edema, swelling and so on)	87	23.51	149	6.75	0.42

### Ranking, informant consensus factor and multipurpose of medicinal plants

Among all ailments in the villages surveyed, traumatic injury was the most commonly disease against which a high number of medicinal plants (67 species) were prescribed. Seven medicinal plant species were used effectively for treating traumatic injury according to key informants. The results revealed *Gaultheria leucocarpa* was the most preferred medicinal plant, followed by *Acanthopanax trifoliatus*, and *Sargentodoxa cuneata* (Table 5).

Table 4 gave an overview of the main illness categories. The diseases that were prevalent in the study area had relatively higher F_IC_ values. Medicinal plants to treat certain disease effectively and with reputation in Maonan communities also have higher F_IC_: traumatic injury and sprain (0.74), fever and malaria (0.73) and infantile diseases (0.75). Moreover, informants indicated the effectiveness of traditional medicines to get relief from certain diseases including traumatic injury, bone fracture, health problems associated with the liver disorder, snake bite, and spider poisoning.

**Table 5:** **Preference ranking to medicinal plants used to treat traumatic injury**

**List of medicinal plants**	**Informants**								**Total**	**Rank**
	**R**_**1**_	**R**_**2**_	**R**_**3**_	**R**_**4**_	**R**_**5**_	**R**_**6**_	**R**_**7**_	**R**_**8**_		
*Acanthopanax trifoliatus*	4	7	5	5	7	5	4	5	42	2
*Bauhinia championii*	3	4	6	7	1	3	1	2	27	5
*Gaultheria leucocarpa*	5	5	7	6	5	6	6	3	43	1
*Justicia ventricosa*	2	6	3	1	4	4	2	4	26	6
*Polygonum chinense*	6	1	1	2	2	1	3	1	17	7
*Sargentodoxa cuneata*	7	3	4	4	3	7	5	7	40	3
*Sambucus williamsii*	1	2	2	3	6	2	7	6	29	4

Key--R represented respondents; Scores in the table indicated ranks given to medicinal plants based on their scarcity. Highest number (7) is for the medicinal plants which informants thought most preferred in the area and the lowest number (1) for the least preferred medicinal plant.

The Maonans naturally relied on plants for multipurpose. Table 6 showed the most frequently inventoried medicinal plants had more functions used by the Maonans in local societies. In addition to medicinal value, most of medicinal plants were also valued for their economic, edible and ornamental values which were considered to serve an ecological role in the study sites. These plants included *Acanthopanax trifoliatus*, *Litsea pungens*, *Platycodon grandiflorus*, *Rubus parvifolius*, and *Talinum paniculatum*. Besides their medicinal purpose, these plants were sold in the local markets for the purposes of foods, spices and herbal teas, such as *Allium fistulosum*, *Allium tuberosum*, *Cinnamomum cassia*, *Perilla frutescens*, *Oenanthe javanica*, *Gardenia jasminoides*, *Houttuynia cordata*, and *Juglans regia*.

**Table 6:** **Most frequently inventoried medicinal plants**

**Species name**	**Medicinal value**	**Edible value**	**Economic value**	**Ornamental value**
*Acanthopanax trifoliatus*	√	√	√	√
*Buddleja officinalis*	√	√	√	
*Houttuynia cordata*	√	√	√	
*Litsea pungens*	√	√	√	√
*Murraya exotica*	√		√	√
*Nephrolepis cordifolia*	√		√	
*Paederia scandens*	√	√		
*Platycodon grandiflorus*	√	√	√	√
*Rauvolfia verticillata*	√		√	√
*Rubus parvifolius*	√	√	√	√
*Sargentodoxa cuneata*	√		√	
*Talinum paniculatum*	√	√	√	√
*Tetrapanax papyrifer*	√		√	

### Mode of preparation, condition, dosage of application

Various plant species were collected and used immediately. Most of the medicinal formulations were administrated orally in ailment categories other than dermatological problems. In dermatological ailments, plants were administrated externally. Water and some additives were often used in the preparation of remedies, such as alcohol, oil, honey, salt, sugar, eggs, chicken, duck and meat. The additives were claimed to either increase nutrition or improve flavor. Most informants used measuring units such as cup, bowl, spoon, fingers and scale but still differed in the doses they administered. The various ways of measuring dosage were generally categorized under three major classes. One dosage was used for those medicinal plants which were expected to be highly toxic. For such medicines the measurement was undertaken by number or weight. The second was the dosage used for medicinal plants which have side effect. The dosage was measured by their hand and taken by container. The third case referred to the medicinal plants without any observable side effects. Medicines prepared were taken according to patients’ personal preference.

Most of the medicinal plant preparations involved the use of single plant species or a single plant part while those mixing different plants or plant parts were less encountered in the study area excluding those for treating bone fracture, rheumatism and other difficult diseases. Suffering from common diseases (common cold, indigestion, mosquito bite and so on), the Maonans usually picked up some medicinal plants for treatments by themselves. Otherwise, they should turn to the Maonan healers for help, and the local healers usually prepared remedies by mixing various plants or plant parts. Lack of consistency regarding amount of medicines was observed among informants. There was no concise standard in measurement or unit used among the informants.

### Threats to medicinal plants and conservation practices

Various factors that were considered as main threats for medicinal plants were recorded by discussion with the informants in the study area. The principal threats of medicinal plants were reported to include drought, deforestation, medicinal purpose, and firewood collection in this area. Informants ranked that the major factors were deforestation for the purpose of agricultural expansion (75%), drought (10%), collection of medicinal plant material (10%) and fire wood (5%). The Maonan people knew the benefits of conserving medicinal plants. However, the effort of conserving medicinal plants was very limited, because most medicinal plants were collected from wild. Even the local healers who frequently made use of medicinal plants for livelihood did not conserve medicinal plants very well, and they preferred to collect them from wild when using for patients.

## Discussion

### Medicinal plants and associated traditional knowledge

On the basis of field investigation and literature studies, 368 species of medicinal plants belonged to 295 genera and 115 families were cataloged. Chinese name, scientific name, local name, family name, used parts and the treatment of diseases were listed. Asteraceae (with 24 species) occupied the highest proportion (6.52%), followed by Fabaceae, Rosaceae and Euphorbiaceae. Moerman also found that species of plants in the sunflower family (Asteraceae) tended to be represented in ethnobotanical usage in excess of what would be expected by their occurrence in local environments [27-29]. In contrast, Moerman identified the greater number of families across North America in general. The most widely used plant remedies by the Maonans were obtained from herbaceous species which constituted the highest category of 203 species (55.16%). Similar findings were reported by other studies throughout the world, and the authors reported that people derived their medicine from herbs partly because of the fact that forests had been degraded, and it took less time and effort to harvest plant material from medicinal herbs [6,30-32].

The special geographical environment results in the rich biodiversity of medicinal plants in the study area. The Maonans have learnt to use local medicinal plants for treatment and prevention in the course of struggling with the ailments. The number of reported medicinal plants and their uses by the Maonans indicate the depth of indigenous knowledge on the medicinal plants and their applications. The Maonans have collected their indigenous knowledge and experience of medicinal plants. Without written language, the knowledge of medicinal plants is still taught orally in the Maonan communities. There is not data record or any illustrated identification which guides for the medicinal plants of Maonan people and their uses.

The Maonans have the traditional customs of disease prevention and emphasize on the function of medicinal food in ordinary life. They usually add medicinal plants into food for the purpose of enhancing the body’s immunity and disease resistance, such as *Talinum paniculatum*, *Gymnotheca chinensis*, *Osbeckia opipara* and so on. The Maonans have the custom of collecting the medicinal plants for cooking and bathing in dragon-boat festival, such as *Acorus calamus*, *Curcuma longa*, *Paederia scandens* and *Leonurus artemisia*. They believe that it would be beneficial for their health. This is because many plants matured in the season of dragon-boat festival [33].

### Preparation, dosage and route of administration of medicinal plants

The most widely harvested part was the whole plant, followed by the roots, leaves, stems and others. The Maonan people used a lot of roots, stems, rhizomes and bark for medicinal purpose. They believed these parts were the most effective. However, such collection of the medicinal plants might kill or damage plants when harvesting. Utilization of leaves might not cause detrimental effect on the plants compared with plant species that root was utilized. Most of medicinal plants were claimed to be prepared from a single species or plant part in the present study, and the different parts of medicinal plant were used to treat disparate diseases. Although Maonan people preferred to treat illnesses with single species, it was observed that the healers mostly used multiple species or plant parts in order to increase the function and efficacy of the drug as they reported during the interviews. Representatively, the Maonan healers mostly used more than one plant species to prepare remedy for treating bone fracture and traumatic injury.

Grinding was the most widely used method of preparation for remedy in the study area. Pounding and powdering fresh plant materials were the other methods of preparation in the study area. Due to the efficiency and richness of the fresh medicinal plants in the study area, preference of application of fresh plant parts was observed. Moreover, internal and skin diseases were more prevalent in the study area. The fresh material use might be an attempt not to lose volatile oils, the concentration of which could decrease on drying. Moa *et al.* reported that the disadvantage was that utilization of fresh plant parts may threaten the plants through frequent collection including in dry seasons since local people made minimal efforts in storing dried plant material for later use [6].

The Maonans usually use the processing methods such as decoction, medicinal liquor, external application and medicated bath. The way of using herbs was benefit for the popularity in a simple and easy method. They used different additives like alcohol, oil, honey, salt, sugar, eggs, chicken, duck and meat in order to increase the flavor, taste and general acceptability of certain orally administered remedies. Because of poverty, eating animal meat and eggs could increase proteins and might be helpful for body recovery when the Maonans were ill. The Maonan healers considered that alcohol could promote the blood circulation and accelerate the absorption of exudates. In addition, the Maonan healers used different procedures to administer the medicinal plants and alcohol combinations. The medicinal plants were soaked in alcohol for nearly one month and then the patients could drink or applied externally on the affected parts. For example, *Acanthopanax gracilistylus*, *Achyranthes bidentata*, *Ardisia gigantifolia*, *Ardisia japonica*, *Arisaema heterophyllum*, *Davallia mariesii*, *Dipsacus asperoides*, *Drynaria propinqua*, *Homalomena occulta*, *Sambucus williamsii*, *Bauhinia championii*, *Murraya exotica*, and *Paris polyphylla* were usually soaked in alcohol for treating traumatic injury and bone fracture.

### Effectiveness and popularity of medicinal plants

Due to the influence of geography, climate and food culture in Maonan areas, the Maonan healers understood the varieties of diseases, such as traumatic injury, snake bite, hepatitis, respiratory disease, digestive system disease, rheumatoid arthritis, and skin problems. The local people expressed they preferred to use traditional medicines rather than western drugs to get relief from some diseases including bone fracture, health problems associated with the liver, snake bite and those caused by hepatitis. The Maonan healers treated ailments based on the patients’ physical conditions, lack of consistency regarding amount of medicines to be used was observed among informants during the interviews. The healers usually did not know which ingredients were important for the therapeutic effect in the multiple prescriptions. The lack of precise dosage was one shortage of traditional medicinal plant uses.

Most of Maonan people knew how to use several medicinal plants for treating ailments and health protection. Traditional medicine knowledge was not only in the hands of the Maonan healers and herbalists in the study area. Moreover, Maonan people grew medicinal plants in their home gardens. Plant species maintained by Maonan healers was found to be significantly distinct from plant species managed by farmers. The Maonan healers knew more than 30 medicinal plant species, while most of the non-healers reported less than 15 species. Ethnomedicinal usage of plants managed by healers was remarkably distinct from usage categories managed by farmers. The Maonan healers were reported to use a combination of multiple medicinal plants to treat an illness, but the farmers always used single plant species or a single plant part.

### Medicinal plant cultivation and trade

The Maonan people in the study area knew the benefits of conserving medicinal plants. However, the effort of conserving medicinal plants was very limited. For example, only 20.75% of medicinal plants were collected from home gardens, and most of the plants from home gardens were used for foods, spices and substitutes for tea. The majority of medicinal plants were harvested from wild habitats. Even Maonan healers who made use of medicinal plants for a livelihood did not conserve the important medicinal plants in their home gardens, and they preferred to collect them from wild or hidden places when patients visited them. It was explained by informants that local healers did not let the other villagers know the identity of the medicinal plants they were using. Informants further explained that if healers planted the species in their home gardens, they worried that somebody else might recognize them when they were preparing the medicine from the plants. Thus the healers’ income would be decreased.

Because of complex terrain and language barrier, the Maonans have been in the traditional self-sufficient agricultural economy in the karst areas. There is a seasonal medicinal market which opens 3 times each month. The sites of purchase and sale of local medicinal plants are located in the town. The medicinal plants grown by farmers were used for household healthcare and little was sold in herbal markets, while medicinal plants were cultivated by healers rarely for trading, either. Not many medicinal plants were solely cultivated for their medicinal purpose, except that the plants were multipurpose (Table 6). Lack of water and land, most Maonan people would prefer to cultivate foods or cash crops rather than medicinal plants. The other reason was that most medicinal plants were not sold at reasonable prices and therefore not profitable, providing very little incentives for their cultivation. The local medicinal markets were small-scaled and were not paid enough attention. The markets provided convenience for the exchange of local medicinal plants, but not providing a good place for indigenous knowledge. This trend might not be beneficial for maintaining traditional practices and giving traditional knowledge the respect it deserves.

### Threats to medicinal plants and conservation practices in the study area

According to informants, nowadays it would take a lot of time and travel long distances to search for some medicinal plants, especially trees and some shrubs. The principal threats of medicinal plants were reported in the study area, including deforestation for agricultural purposes, urbanization, drought, over-harvesting of known medicinal species and firewood collection. Also, informants ranked deforestation for agricultural purposes as the most serious threat to medicinal plants followed by drought, collection of other different factors and firewood. The conservation of medicinal plants was less realized in the study area.

### Medicinal plants knowledge secrecy, mode of transfer, gender issue and threats between different social groups within the Maonan area

This study highlighted the rich biodiversity of medicinal plants and ethnomedicinal practice in Maonan area to maintain wellbeing and support livelihoods. This study revealed that, most of the knowledge on herbal remedies was handled down to the younger members of the community by elders orally, who were over 40 years old and less-educated. The Maonan herbalists and healers were male, and only men had the opportunities to study knowledge of traditional medicinal plants in the family. The conservative concept of Maonan healers made a systematic indigenous knowledge of Maonan traditional medicine, which had always been in the hands of a few people. The age structure and knowledge transmission system had the negative influence on the inheritance and development of indigenous knowledge. It dramatically exposed the vulnerability of traditional medicinal knowledge if its transmission was limited by acculturation or inter-ethnic exchange from generation to generation [15,34,35].

Nowadays, the fact is that inheritance of indigenous knowledge is difficulty from the elders to the young generation. Most young people do not believe that studying indigenous knowledge is beneficial for their life because it is less profited compared to working in the urban area. Furthermore, some young people think traditional medicine is anti-science. While male Maonan people work outside, women take responsibility to take care of their families and educate children. If women know how to use medicinal plants, it will be beneficial for training children. According to our interviews, the Maonan women are eager to learn the traditional herbal medicinal knowledge. They may become potential and effective inheritors in the Maonan area, if customary inheriting system allows them.

## Conclusions

The paper is an ethnobotanical study on medicinal plants used by Maonan people. We documented 368 species (belonging to 295 genera and 115 families) of medicinal plants used by the Maonans in Huanjiang Maonan Autonomous County, northern Guangxi, southwest China. These plants were used to treat 95 human diseases, such as traumatic injury, bone fracture, health problems associated with the liver disorder, snake bite, and spider poisoning etc. Traditional knowledge about the use, preparation, and application of these medicinal plants is usually passed verbally from generation to generation. The valuable information about medicinal plants could be preserved while recording in the written form. Moreover, the documentation of medicinal plants can serve as a basis for future investigation of new medicinal resources.

Among the medicinal plant species, the whole plants of herbaceous species are harvested from field and constituted the highest proportion of medicinal plants to be utilized. More roots and barks are used than other plant parts, which imply that traditional medical culture in the Maonan area does threaten some species. Although high numbers of medicinal plant species have been reported to be used for human health problems, many wild species are being threatened by various anthropogenic factors while conservation efforts are less practiced in the study area. Deforestation for agricultural purposes is the major threat factor. To save and protect medicinal plants, the external help is necessary, by providing the Maonan people with both seedlings or seeds and cultivation techniques of medicinal plants.

The Maonan men are the only inheritors to transmit traditional medicinal knowledge to the next generations. Unfortunately, the knowledge on herbal remedies is held by elders, who are less educated and above 40 years old. Most young men prefer to look for jobs in urban areas instead of studying the Maonan’s medicinal knowledge. It is urgent to find solution of conserving and transmitting the traditional medicinal knowledge in the study area.

Thus, government agencies should encourage the Maonan people to maintain the biodiversity and the ethnomedicinal knowledge by providing the local people with planting materials of the most threatened and preferred medicinal and multipurpose species so that they can grow these plants in their home gardens or farmlands. Public awareness is needed to encourage the local Maonan people to sustainably utilize and manage the medicinal plant resources. *Ex situ* and *in situ* conservation measures should be taken to protect the medicinal plants in the study areas from further loss.

### Consent

Permissions were provided by all participants in this study, including the local Maonan people. Consent was obtained from the local communities prior to the field investigations. The authors have all copyrights.
